# Synthesis of C_70_-fragment buckybowls bearing alkoxy substituents

**DOI:** 10.3762/bjoc.16.66

**Published:** 2020-04-15

**Authors:** Yumi Yakiyama, Shota Hishikawa, Hidehiro Sakurai

**Affiliations:** 1 Division of Applied Chemistry, Graduate School of Engineering, Osaka University, 2-1 Yamadaoka, Suita, Osaka 565-0871, Japan

**Keywords:** buckybowl, C_70_, rearrangement through C–H bond activation

## Abstract

Buckybowls bearing a C_70_ fragment having two alkoxy groups were synthesized and their structural and optical properties were investigated by single crystal X-ray analysis and UV–vis spectroscopy. In the synthesis of dioxole derivative **5b**, the regioisomer **5c** was also produced. The yield of **5c** was increased by increasing the reaction temperature, indicating that the rearrangement might involve the equilibrium between the Pd(IV) intermediates through C–H bond activation.

## Introduction

**Figure 1:**
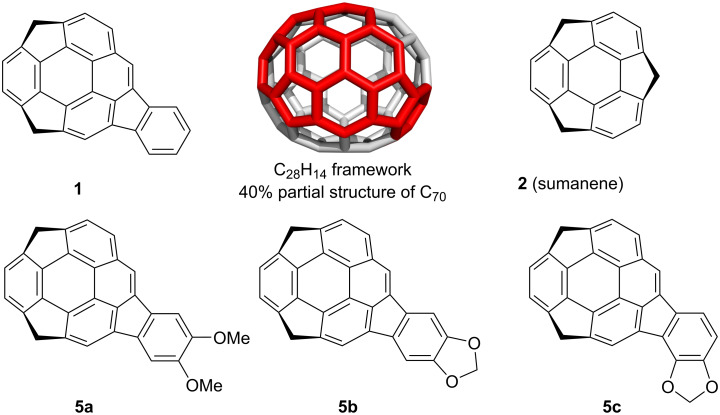
Structure of the target buckybowls **5a–c**.

The study of buckybowls, the bowl-shaped π-conjugated aromatic hydrocarbons corresponding to the fragments of fullerenes, pioneered by the works on colannulene and sumanene, have been attracting great interests owing to their unique chemical and physical properties [1–8] and was extended to larger systems [9–17]. Among them, buckybowls having a C_70_ fragment are expected to exhibit different properties from that with C_60_ fragment because most of them consists of acene and/or pyrene units, which might give unique photochemical and electrochemical properties. Recently, we synthesized a buckybowl C_28_H_14_ **1**, which is corresponding to a 40% fragment of C_70_, from C_60_-fragment sumanene (**2**) in three steps via ring expansion by Wagner–Meerwein rearrangement, followed by Pd-catalyzed annulation (Figure 1) [18]. An UV–vis spectroscopy study revealed that the electronic character of **1** rather resembled that of an indenopyrene moiety than that of benzopyrene. Our synthetic route allows to easily introduce substituents on the external aromatic ring of the indenopyrene using various types of *o-*bromo arylaldehydes. Related to our study on buckybowl-containing liquid crystals [19], we planned to introduce alkoxy groups on the **1** framework. Here, we report the synthesis and characterization of dimethoxy derivative **5a** and dioxole derivative **5b** together with an unexpected regioisomer **5c**.

## Results and Discussion

### Synthesis of dialkoxides **5a–c**

**Scheme 1:**
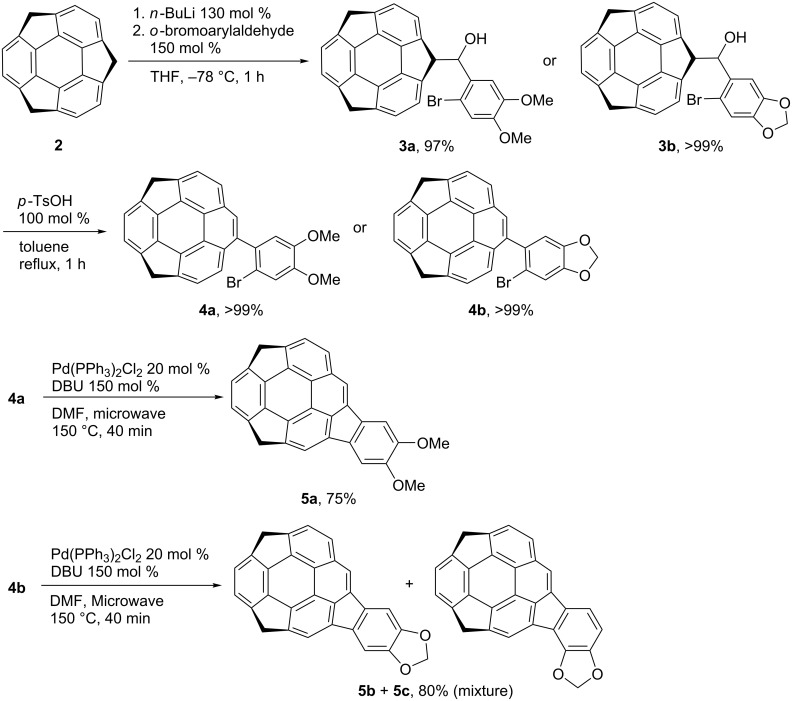
Synthesis of dialkoxides **5a**–**c**.

Dialkoxides **5a**–**c** were prepared according to the previous report on the synthesis of **1** (Scheme 1) [18,20]. The benzylic carbanion generated by the addition of 130 mol % *n*-BuLi to **2** in THF at −78 °C was treated with 150 mol % of the corresponding arylaldehydes to afford **3a** and **3b** quantitatively. The Wagner–Meerwein rearrangement from **3a** and **3b** to **4a** and **4b** by 100 mol % of *p*-TsOH in toluene under reflux conditions also occurred quantitatively. The final cyclization of **4a** was carried out using 20 mol % of Pd(PPh_3_)_2_Cl_2_ and 150 mol % DBU in DMF at 150 °C under microwave irradiation conditions to afford the desired dimethoxy derivative **5a** in 75% yield. In contrast, when the reaction of **4b** was performed, not only the desired product **5b** but also the unexpected regioisomer **5c** was obtained. The temperature dependency of the product ratio between **5b** and **5c** was investigated and the results are shown in Table 1. The cyclization did not proceed under 140 °C, and at 140 °C the total yield is low (41% after 40 min microwave irradiation) but the ratio of **5b** was the highest (**5b**/**5c** = 10:1). The reaction efficiency was high at 150 °C to reach 80% total yield, and the ratio of **5b**/**5c** was 10:3. By increasing the temperature, the ratio of **5c** was increased although the total yield was decreased. It should be noted that the conversion between **5b** and **5c** under the same conditions was not observed.

**Table 1:** The change of **5b**/**5c** ratio in the product mixture at various temperatures.

reaction temp. (°C)	yield (%)	**5b**/**5c**

140	41	10/1
150	80	10/3
160	67	10/4
170	77	10/5
180	50	10/7

**Scheme 2:**
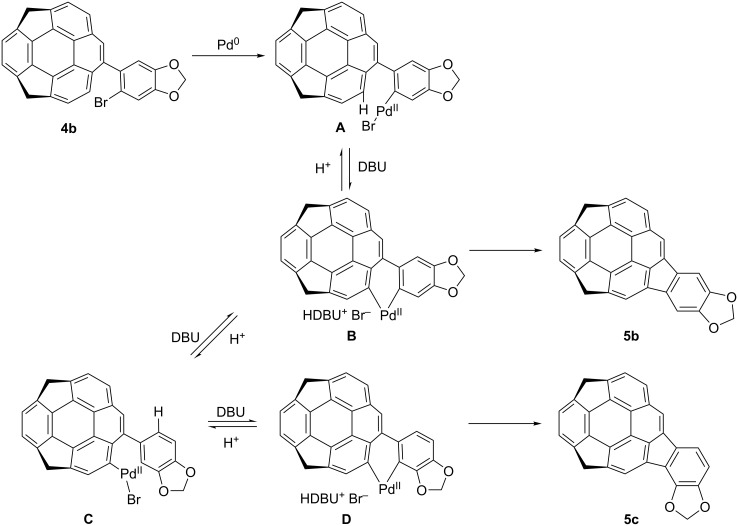
Proposed mechanism of the formation of **5b** and **5c**.

The above results strongly suggested the existence of an equilibrium between the intermediates corresponding to products **5b** and **5c**. A possible mechanism is shown in Scheme 2 [21–25]. After the oxidative addition of **4b** to Pd^0^ to generate intermediate **A**, the neutral palladium(II) intermediate **B** is formed. Two competitive processes, the reductive elimination from **B** to give the product **5b**, and the 1,5-palladium migration from **A** to **C** through **B**, might exist, and from **C**, after the bond rotation, the intermediate **D** would form to afford the isomer **5c**. The selectivity of these two processes are dependent on the temperature as shown in Table 1. It is assumed that the ring-rotation process, which generated the regioisomer did not occurred in case of **4a** because of two larger methoxy groups than the methylenedioxy group.

### Crystal structures of **5a–c**

**Figure 2:**
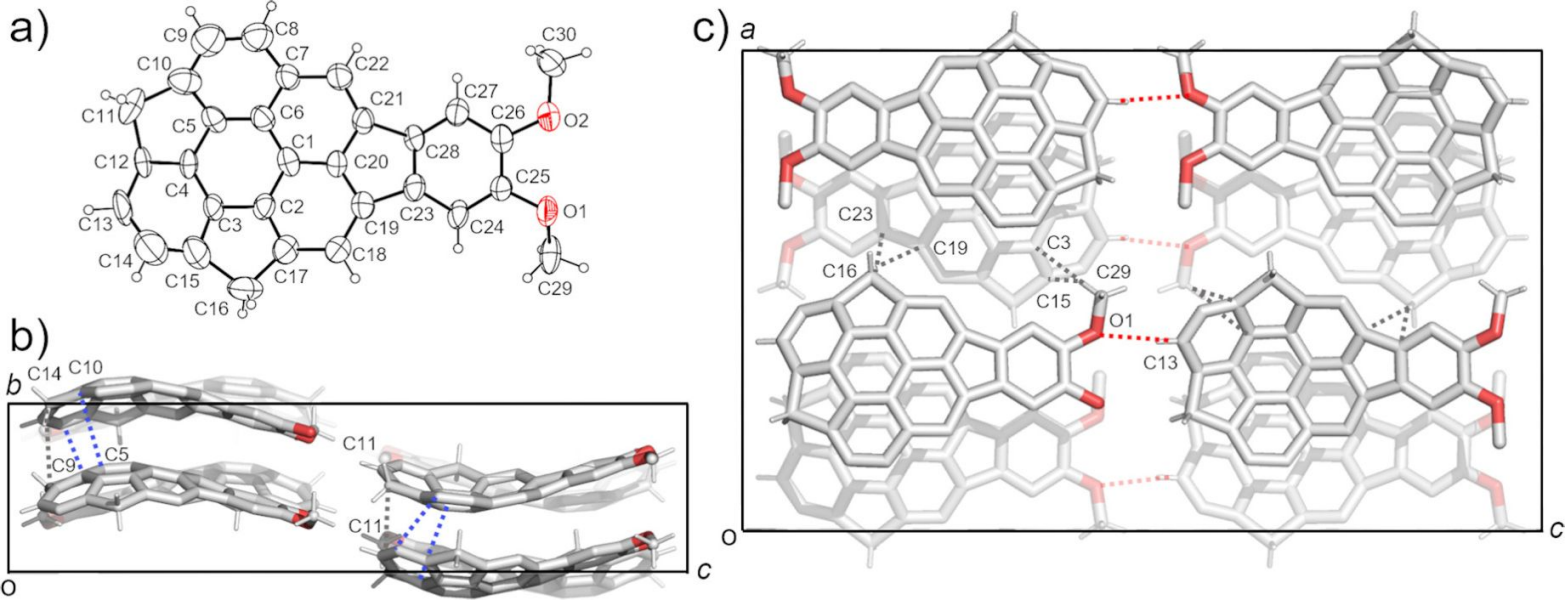
Crystal structure of **5a**. a) ORTEP drawing of the crystallographically independent unit with thermal ellipsoid at 50% probability b) Packing structure viewed from the *a* axis and c) from the *b* axis. The dotted lines indicate; blue: π–π, grey: CH···π, red: CH···O interactions. In b) and c), hydrogen atoms which are not engaged in any interactions are omitted for clarity.

Single crystals of **5a**–**c** were successfully obtained by vapour diffusion method using CHCl_3_/hexane conditions. Figure 2 shows the crystal structure of **5a**. The crystal was obtained as a racemic compound containing a pair of two enantiomers defined by bowl chirality [26], as a result of the rapid bowl inversion under the crystallization conditions. **5a** formed a columnar structure with alternative stack in convex-to-concave manner along the *b* axis with the overlap of the half part of the bowl structure (Figure 2b). All the columns along the *a* axis possessed the same stacking direction, while the neighboring columns along the *c* axis were in opposite directions (Figure 2c). Although the relatively low diffraction data quality prohibited the detailed discussion about the interaction distances, both π–π (C9∙∙∙C14, C5∙∙∙C10) and CH∙∙∙π (C11∙∙∙C11) interactions were confirmed within the column. These columns were further connected with the neighboring columns which possessed the same stacking direction (along the *a* axis) by CH∙∙∙π interactions (C16∙∙∙C23, C16∙∙∙C19, C29∙∙∙C3, C29∙∙∙C15), while connected to the columns with opposite stacking direction via CH∙∙∙O type weak hydrogen bonds (C13∙∙∙O1) along the *c* axis (Figure 2c).

**Figure 3:**
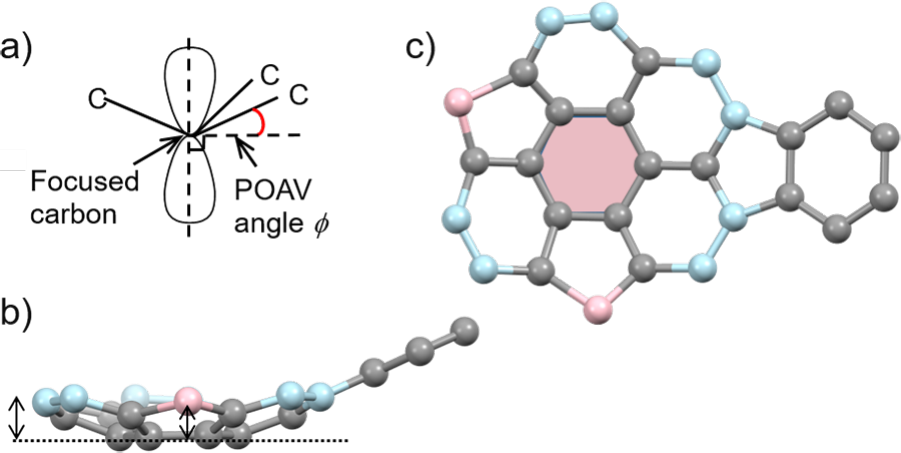
a) Definition of POAV angle (φ). b) Side and c) top view of the molecular skeleton of **1**. The double-headed arrow show the perpendicular line from the peripheral carbons to the bottom hexagonal ring coloured in c). In b) and c), pink colored atoms are benzylic, and blue colored ones are aromatic carbons.

**Figure 4:**
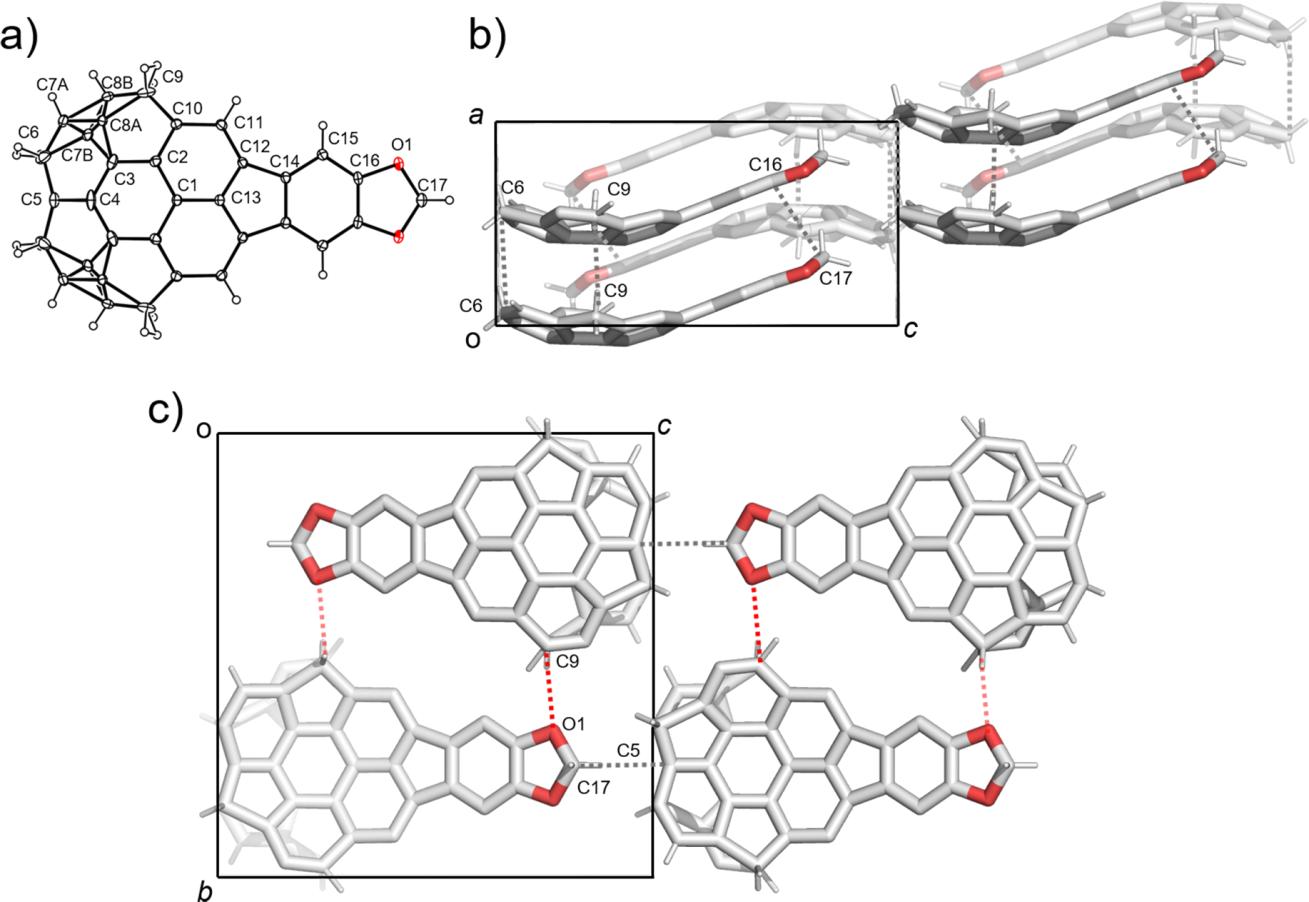
Crystal structure of **5b**. a) ORTEP drawing of the crystallographically independent unit with thermal ellipsoid at 50% probability. b) Packing structure viewed from the *b* axis and c) from the *a* axis. The dotted lines indicate; grey: CH···π, red: CH···O interactions. In b) and c), hydrogen atoms which are not engaged in any interactions and the contribution of the one enantiomer are omitted for clarity.

**5b** also gave the mixture of the two enantiomers, however, they were disordered with 50% site occupancy (Figure 2a). The POAV (π-orbital axis vector) pyramidalization angle φ [27], which is often used for quantifying the curvature of curved π-conjugated materials (Figure 3a) showed 6.5° at C1 as the minimum value, and 7.6° at C3, which is surrounded by two hexagonal rings and one pentagonal ring, as the maximum value, while the none-substituted **1** shows 6.2° and 7.6°, respectively (Table 2) [18]. Bowl depths, defined by the length of the perpendicular lines (Figure 3b, double-headed arrow) from its peripheral carbons to the bottom hexagonal ring’s plane (Figure 3c, red coloured part) in **5b** were 0.80–0.84 Å from the peripheral benzylic carbons and 0.80–0.89 Å from the peripheral aromatic carbons, respectively, while 0.74–0.79 Å and 0.79–0.99 Å in **1**, respectively (Table 2) [18]. As observed in the crystal of **5a**, **5b** formed convex-to-concave type stacking columns along the *c* axis while the stacking mode was eclipsed manner, in which molecular skeletons were completely overlapped (Figure 4b,c). The stacking directions of the columns were alternatively changed along the *b* axis. Unlike **5a**, the stacking columns in **5b** were exclusively stabilized by CH∙∙∙π interactions (C6∙∙∙C6: 3.77 Å, C9∙∙∙C9: 3.77 Å, C16∙∙∙C17: 3.51 Å) (Figure 4b). These columns were further connected to the neighboring columns by CH∙∙∙O type hydrogen bonds (C9∙∙∙O1: 3.32 Å) along the *b* axis and CH∙∙∙π interactions (C17∙∙∙C5: 3.60 Å) along the *c* axis (Figure 4c).

**Table 2:** Experimental POAV angles and bowl depths of **5b** at the specific focused carbons.

molecule	POAV angle φ/°				bowl depth/Å					
		
					benzylic		aromatic			

**5b**	C1 C2 C3a	6.5 6.7 6.7	C3b C4	7.6 7.3	C6 C9	0.84 0.80	C6 C7A C8B	0.81 0.89 0.88	C9 C11 C12	0.80 0.89 0.84

^a^Calculated using 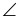
C4–C3–C8A, 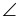
C8A–C3–C2, 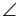
C2–C3–C4; ^b^calculated using 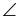
C4–C3–C7B, 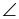
C7B–C3–C2, 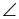
C2–C3–C4.

**Figure 5:**
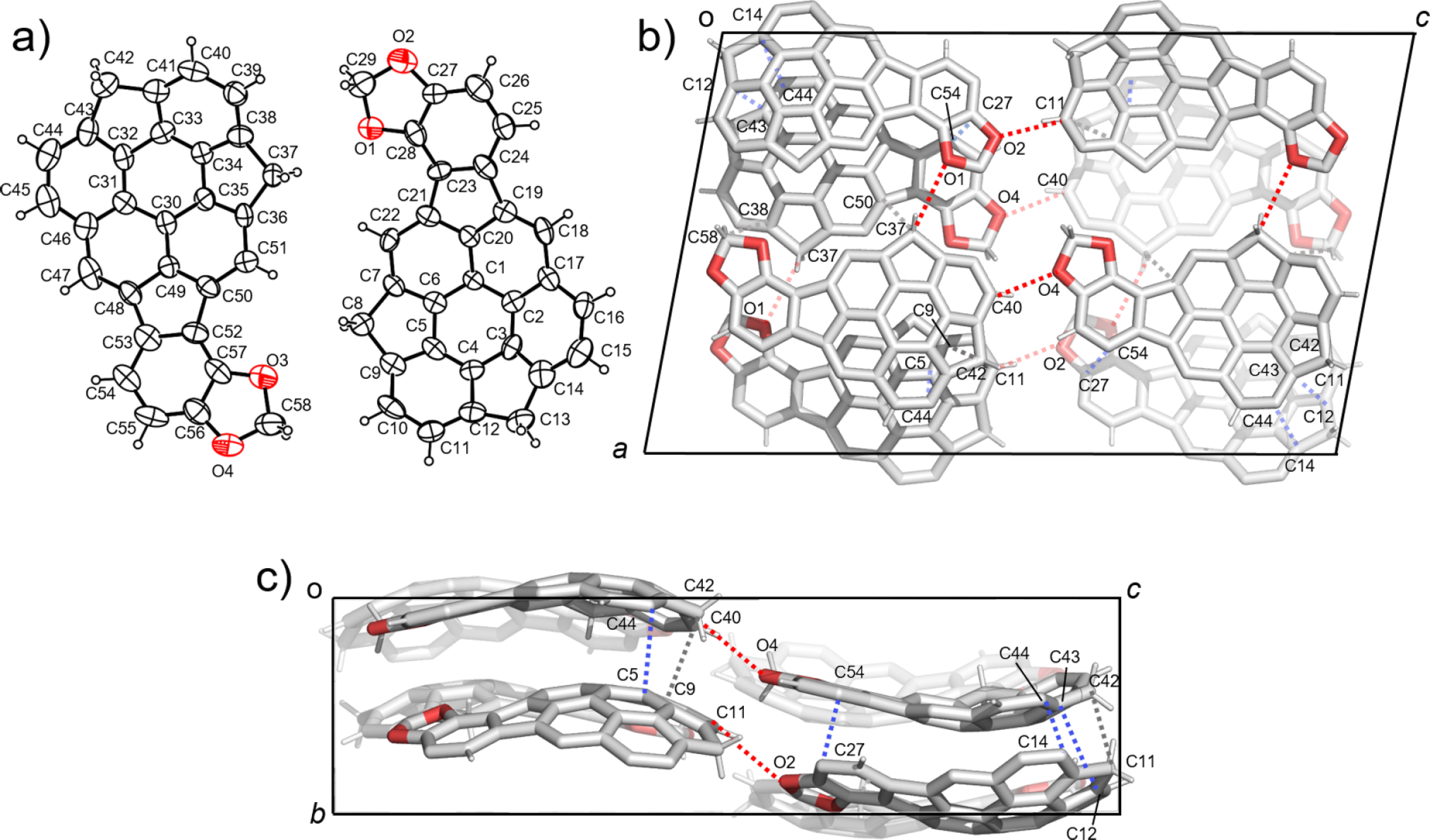
Crystal structure of **5c**. a) ORTEP drawing of the crystallographically independent unit with thermal ellipsoid at 50% probability b) Packing structure viewed from the *b* axis and c) from the *a* axis. The dotted lines indicate; blue: π–π, grey: CH···π, red: CH···O interactions. In b) and c), hydrogen atoms which are not engaged in any interactions are omitted for clarity.

In the crystal structure of **5c**, two crystallographically independent units were observed (Figure 5a). **5c** also contained both of the enantiomers and formed a columnar structure along the *b* axis with the slipped stack manner, which was composed of only one side of the enantiomer (Figure 5b,c). The columns with the same stacking direction were arranged along the *a* axis, while an alternative stacking direction was observed along the *c* axis. Although relatively low diffraction data quality prohibited the detailed discussion about the interaction distances, the stabilization of the 1-dimensional stacking column of **5c** by both π–π (C5∙∙∙C44, C27∙∙∙C54, C14∙∙C44, C12∙∙∙C43) and CH∙∙∙π (C11∙∙∙C42, C42∙∙∙C9) was clearly observed (Figure 4b c). As found in the other two, the stacking columns in **5c** crystal were also further connected each other by both CH∙∙∙π interaction (C58∙∙∙C38, C37∙∙∙C50) and CH∙∙∙O type hydrogen bonds (C37∙∙∙O1, C11∙∙∙O2, C40∙∙∙O4) (Figure 5c).

**Figure 6:**
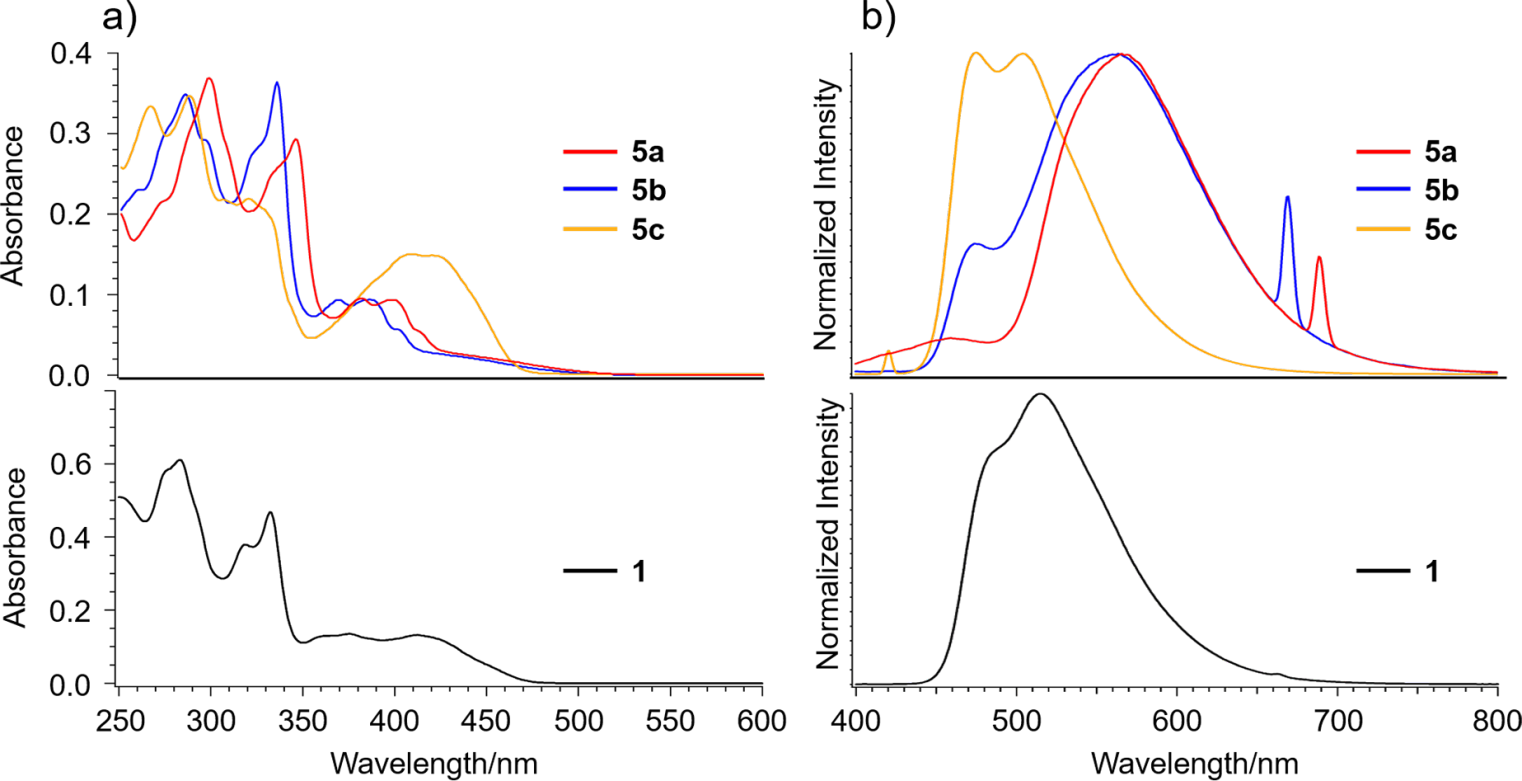
a) UV–vis spectra and b) emission spectra of **1** and dialkoxides **5a**–**c**. For all the spectra, the concentration was 1 × 10^−5^ M in CH_2_Cl_2_. Excitation wavelength: 332 nm for **1**; 345 nm for **5a**; 335 nm for **5b**; 319 nm for **5c**.

Photophysical properties of the dialkoxides were investigated by UV–vis and emission spectroscopies (Figure 6). UV–vis spectra of **5a** and **5b** well reflected the electric property of **1**, showing two strong bands observed at around 280–300 nm and 330–350 nm, and a broad one at around 350–480 nm, which was attributable to the indenopyrene moiety of **1** (Figure 6a) [18]. Meanwhile, **5a** and **5b** showed emission bands at 564 nm and 566 nm, respectively, which were red shifted around 50 nm from that of **1**, clearly indicating the effect of the introduction of dialkoxides (Figure 6b). In contrast, **5c** exhibited different features in both UV–vis and emission spectra from the other two. In the UV–vis spectrum of **5c**, the splitted sharp absorptions at 266 and 287 nm and a broad band at 320 nm together with a relatively strong broad band at 409 nm are visible. The emission spectrum of **5c** was similar to that of **1** rather than those of **5a** and **5b**. These differences indicate the substitution position of the dialkoxides significantly affected the electric nature of the molecules even though **5a**–**c** possess the same molecular skeleton of **1**.

## Conclusion

As described above, we succeeded in synthesizing three different alkoxy-substituted C_70_-fragment buckybowls **5a**–**c**. In particular, **5c** was not an intended molecule, but was formed unexpectedly through the rearrangement through the Pd-catalyzed C–H bond activation reaction. The X-ray crystal structure analysis of **5a**–**c** clearly revealed there nature in the solid state to form a 1-dimensional columnar structure stabilised by π–π and/or CH∙∙∙π interactions with full or partial overlap of the molecular skeleton as seen in the crystal structure of **1**, however, each packing fashion is different depending on the substituent. UV–vis and emission spectra of **5a**–**c** well showed the effect of the introduction of the dialkoxides onto the skeleton of **1**, in which the substitution position also contributed to their electric properties. These results give us a lot of suggestions for further investigations to design buckybowl-containing liquid crystals [19].

## Experimental

### General

All experiments with moisture- and air-sensitive compounds were performed in anhydrous solvents under nitrogen atmosphere in flame-dried glassware. All reagents were purchased from commercial sources and used without further purification unless otherwise noted. The microwave experiment was carried out with a Biotage Initiator Eight EXP. UV–vis absorption spectra were recorded on a JASCO V-670 spectrometer and a SHIMADZU UV-1800 spectrometer. Fluorescence spectra were recorded on a JASCO FP-6500 spectrometer. Melting points were determined on a Stanford Research Systems MPA 100 or a Yanako MP-500P apparatus and were uncorrected. Infrared (IR) spectra were recorded on a JASCO FT IR-4100 spectrometer using dispersed KBr pellets. ^1^H and ^13^C NMR spectra were measured at 23 °C on a JEOL RESONANCE JNM-ECZ400S spectrometer at 400 MHz and 100 MHz, respectively. CDCl_3_ was used as a solvent and the residual solvent peaks were used as an internal standard (^1^H NMR: CDCl_3_ 7.26 ppm; ^13^C NMR: CDCl_3_ 77.00 ppm). High-resolution fast atom bombardment (FAB) mass spectra were measured on a JEOL JMS-700 spectrometer. TLC analysis was performed using Merck silica gel 60 F_254_, and the preparative TLC (PTLC) purification was conducted using Wakogel B-5F PTLC plates. Elemental analyses were measured on a J-Science Micro corder JM10 at the Analysis Center at Osaka University.

### General experimental procedure for the addition reaction

In a similar manner as described in [18], to a solution of **2** (0.20 mmol) in dry THF (20 mL) was added dropwise *n*-BuLi in hexane solution (0.26 mmol) at −78 °C. After stirring for 10 min, to the reaction mixture was added arylaldehyde (0.30 mmol) at −78 °C. The mixture was stirred for 1 h, warmed up to room temperature and quenched by sat. NH_4_Cl_aq_ The resulting mixture was extracted with CH_2_Cl_2_. The extract was washed with brine, dried over anhydrous Na_2_SO_4_, filtered and concentrated under reduced pressure. The mixture was purified by PTLC (ethyl acetate/hexane = 3:2 for **3a,** ethyl acetate/hexane = 1:4 for **3b**) to afford **3**.

### General experimental procedure for the rearrangement reaction

In a similar manner as described in [18], to the mixture of **3** (0.19 mmol) and *p*-toluenesulfonic acid (0.19 mmol) was added a dry toluene (20 mL). The mixture was refluxed with stirring for 1 h. After the addition of sat. NaHCO_3aq_, the mixture was extracted with CH_2_Cl_2_. The extract was washed with brine, dried over anhydrous Na_2_SO_4_, filtered and concentrated under reduced pressure. The mixture was purified by PTLC (CH_2_Cl_2_/hexane = 4:1 for **4a**, CH_2_Cl_2_/hexane = 3:2 for **4b**) to afford **4**.

### Preparation of dialkoxides by intramolecular coupling reaction

In a similar manner as described in [18], in a glove box to the microwave vial was added **4** (0.030 mmol), Pd(PPh_3_)_2_Cl_2_ (6.0 μmol), and degassed DMF (3 mL) and prior sealing the vial DBU (6.7 μL, 0.045 mmol) and the mixture was stirred for 40 min at 150 °C using microwave. After the addition of ethyl acetate/hexane, the mixture was washed with water (3 times) and brine (3 times), dried over anhydrous Na_2_SO_4_, filtered and concentrated under reduced pressure. The mixture was purified by PTLC (only CH_2_Cl_2_ for **5a**, CH_2_Cl_2_/hexane = 1:3 for **5b** and **5c**) to afford both **5** as yellow solid.

### Characterization data

**3a**: yellow solid; yield: 97% (98.3 mg (0.19 mmol) from 52.8 mg (0.20 mmol) of **2**). mp 133 °C; IR (KBr) ν: 3438, 3003, 2922, 2841, 1502, 1398, 1257, 1213, 1159, 1032, 798 cm^−1^; ^1^H NMR (CDCl_3_) δ (ppm) 7.29 (s, 1H), 7.08 (s, 2H), 7.05 (s, 1H), 7.04 (d, *J* = 8.0 Hz, 1H), 7.00 (d, *J* = 8.0 Hz, 1H), 6.81 (d, *J* = 7.6 Hz, 1H), 6.68 (d, *J* = 7.6 Hz, 1H), 5.92 (dd, *J* = 5.6, 3.2 Hz, 1H), 4.67 (d, *J* = 19.4 Hz, 2H), 4.00 (d, *J* = 5.6 Hz, 1H), 3.90 (s, 3H), 3.86 (s, 3H), 3.39 (dd, *J* = 19.4, 4.8 Hz, 2H), 2.76 (d, *J* = 3.6 Hz, 1H); ^13^C NMR (CDCl_3_) δ (ppm) 150.00, 149.87, 148.97, 148.91, 148.86, 148.82, 148.76, 148.74, 148.64, 148.46, 147.91, 147.30, 133.58, 125.81, 124.18, 123.54, 123.46, 123.38, 115.10, 112.33, 111.05, 74.39, 61.75, 56.26, 41.85, 41.84; FAB MS *m*/*z*: [M^+^] calcd for C_30_H_21_BrO_3_, 508.0674; found, 508.0665.

**3b**: yellow solid; yield: >99% (148 mg (0.30 mmol) from 79.2 mg (0.30 mmol) of **2**). mp 133 ºC; IR (KBr) ν: 3546, 3041, 3014, 2889, 1502, 1475, 1398, 1236, 1038, 931, 795 cm^−1^; ^1^H NMR (CDCl_3_) δ (ppm) 7.31 (s, 1H), 7.11 (s, 2H), 7.07 (s, 1H), 7.06 (d, *J* = 7.6 Hz, 1H), 7.05 (d, *J* = 7.6 Hz, 1H), 6.82 (d, *J* = 7.6 Hz, 1H), 6.79 (d, *J* = 7.6 Hz, 1H), 6.07 (d, *J* = 1.6 Hz, 1H), 6.04 (d, *J* = 1.6 Hz, 1H), 5.91 (dd, *J* = 6.4, 3.6 Hz, 1H), 4.70 (d, *J* = 17.6 Hz, 2H), 4.00 (d, *J* = 6.4 Hz, 1H), 3.41 (d, *J* = 19.6 Hz, 2H), 2.74 (d, *J* = 3.6 Hz, 1H); ^13^C NMR (CDCl_3_) δ (ppm) 149.94, 149.89, 148.86, 148.77, 148.73, 148.72, 148.62, 147.86, 147.79, 147.57, 147.40, 135.04, 125.71, 124.18, 123.45, 123.41, 113.00, 112.51, 108.37, 101.92, 74.54, 61.71, 41.82; FAB MS *m*/*z*: [M^+^] calcd for C_29_H_17_BrO_3_, 492.0361; found, 492.0364.

**4a**: yellow solid; yield: >99% (98.5 mg (0.19 mmol) from 98.3 mg (0.19 mmol) of **3a**). mp 186 ºC; IR (KBr) ν: 3018, 2929, 2897, 2839, 1601, 1498, 1437, 1375, 1327, 1244, 1209, 1167, 1020, 791 cm^−1^; ^1^H NMR (CDCl_3_) δ (ppm) 7.92 (d, *J* = 4.0 Hz, 2H), 7.85 (d, *J* = 8.0 Hz, 1H), 7.83 (s, 1H), 7.54 (s, 2H), 7.53 (d, *J* = 8.0 Hz, 1H), 7.25 (d, *J* = 4.0 Hz, 1H), 7.02 (s, 1H), 4.35–4.52 (m, 4H), 4.00 (s, 3H), 3.86 (s, 3H); ^13^C NMR (CDCl_3_) δ (ppm) 149.21, 148.40, 143.94, 143.89, 143.74, 141.20, 141.11, 140.62, 140.46, 139.13, 133.43, 130.55, 130.33, 127.58, 125.81, 125.58, 124.39, 124.20, 124.11, 124.02, 123.23, 115.68, 114.67, 114.31, 56.45, 56.26, 42.14, 42.03; FAB MS *m*/*z*: [M^+^] calcd for C_30_H_19_BrO_2_, 490.0568; found, 490.0569.

**4b**: yellow solid; yield: >99% (86.0 mg (0.17 mmol) from 81.6 mg (0.17 mmol) of **3b**). mp 124 ºC; IR (KBr) ν: 3026, 2897, 1469, 1383, 1223, 1043, 930, 796 cm^−1^; ^1^H NMR (CDCl_3_) δ (ppm) 7.88 (s, 2H), 7.86 (d, *J* = 8.0 Hz, 1H), 7.82 (s, 1H), 7.59 (d, *J* = 8.0 Hz, 1H), 7.49 (s, 2H), 7.31 (s, 1H), 7.03 (s, 1H), 6.12 (s, 1H), 6.11 (s, 1H), 4.46 (d, *J* = 20.2 Hz, 2H), 4.28 (d, *J* = 20.2 Hz, 2H); ^13^C NMR (CDCl_3_) δ (ppm) 148.17, 147.48, 143.91, 143.87, 143.73, 141.14, 141.10, 141.04, 141.01, 140.56, 140.37, 139.07, 134.48, 130.49, 130.22, 127.62, 125.75, 125.54, 124.33, 124.14, 124.06, 123.97, 123.06, 114.94, 113.04, 111.85, 102.10, 53.61, 42.10, 41.99; FAB MS *m*/*z*: [M^+^] calcd for C_29_H_15_BrO_2_, 474.0255; found, 474.0258.

**5a**: yellow solid; yield: 75% (4.9 mg (11.9 μmol) from 7.8 mg (15.9 μmol) of **4a**). mp 281 ºC (dec.); IR (KBr) ν: 2929, 2889, 2831, 1606, 1473, 1392, 1290, 1205, 1163, 1053, 858, 783 cm^−1^; ^1^H NMR (CDCl_3_) δ (ppm) 8.16 (s, 1H), 7.98 (s, 1H), 7.93 (d, *J* = 8.0 Hz, 1H), 7.81 (d, *J* = 8.0 Hz, 1H), 7.57 (s, 1H), 7.47 (s, 1H), 7.39 (d, *J* = 7.6 Hz, 1H), 7.34 (d, *J* = 7.6 Hz, 1H), 4.36 (s, 2H), 4.29 (s, 2H), 4.06 (s, 6H); ^13^C NMR (CDCl_3_) δ (ppm) 149.65, 148.38, 148.32, 147.55, 146.49, 146.01, 144.38, 143.85, 142.90, 138.02, 135.45, 135.32, 134.48, 132.17, 127.44, 127.06, 126.66, 124.92, 124.15, 122.85, 120.67, 118.06, 106.01, 104,79, 56.41, 42.77, 41.67; anal. calcd for C_30_H_18_O_2_(H_2_O)_0.5_: C, 85.90%; H, 4.57%; found: C, 85.75%; H, 4.92%.

**5b**: yellow solid; yield: 80% (6.3 mg (16.0 μmol) from 7.9 mg (20.0 μmol) of **4b**). mp 270 ºC (dec.); IR (KBr) ν: 3041, 3006, 2920, 2887, 1460, 1390, 1286, 1159, 1038, 943, 850, 785 cm^−1^; ^1^H NMR (CDCl_3_) δ (ppm) 8.12 (s, 1H), 7.93 (s, 1H), 7.92 (d, *J* = 8.0 Hz, 1H), 7.80 (d, *J* = 8.0 Hz, 1H), 7.48 (s, 1H), 7.39 (s, 1H), 7.38 (d, *J* = 7.2 Hz, 1H), 7.33 (d, *J* = 7.2 Hz, 1H), 6.07 (s, 2H), 4.34 (s, 2H), 4.28 (s, 2H); ^13^C NMR (CDCl_3_) δ (ppm) 148.32, 148.14, 146.93, 146.53, 146.13, 144.39, 144.34, 143.94, 142.86, 137.59, 136.96, 135.21, 135.01, 134.36, 133.59, 127.39, 127.17, 126.55, 124.94, 124.15, 122.87, 120.94, 118.16, 103.56, 102.51, 101.60, 42.75, 41.66; anal. calcd for C_29_H_14_O_2_(H_2_O)_0.4_: C, 86.73%; H, 3.71%; found: C, 86.74%; H, 3.55%.

**5c**: yellow solid; yield: 80% (as 10:3 mixture with **5b** (reaction temp. 150 °C)). mp 267 ºC (dec.); IR (KBr) ν: 2877, 1647, 1469, 1429, 1236, 1097, 1047, 933, 802 cm^−1^; ^1^H NMR (CDCl_3_) δ (ppm) 8.14 (s, 1H), 8.09 (s, 1H), 7.91 (d, *J* = 8.0 Hz, 1H), 7.81 (d, *J* = 8.0 Hz, 1H), 7.54 (d, *J* = 8.0 Hz, 1H), 7.40 (d, *J* = 8.0 Hz, 1H), 7.36 (d, *J* = 8.0 Hz, 1H), 6.82 (d, *J* = 8.0 Hz, 1H), 6.17 (s, 2H), 4.36 (s, 2H), 4.29 (s, 2H); ^13^C NMR (CDCl_3_) δ (ppm) 148.56, 148.06, 146.54, 146.49, 145.77, 144.46, 144.15, 144.05, 142.86, 142.32, 139.70, 137.81, 135.70, 134.69, 134.47, 131.71, 127.37, 126.88, 126.61, 124.96, 124.13, 123.19, 120.68, 120.60, 115.81, 105.84, 101.66, 42.74, 41.70; anal. calcd for C_29_H_14_O_2_(H_2_O)_0.4_: C, 86.73%; H, 3.71%; found: C, 86.58%; H, 3.50%.

### Single crystal X-ray analysis

The diffraction data for **5a** and **5c** were collected on a Rigaku FR-E Superbright rotating-anode X-ray source with a Mo-target (λ = 0.71073 Å) equipped with a Rigaku RAXIS VII imaging plate as the detector at 150 K in house. The diffraction images processsing and absorption correction were performed by using RIGAKU RAPID AUTO [28].

The diffraction data for **5b** was recorded on an ADSC Q210 CCD area detector with a synchrotron radiation (λ = 0.70000 Å) at 2D beamline in Pohang Accelerator Laboratory (PAL). The diffraction images were processed by using HKL3000 [29]. Absorption correction was performed with the program PLATON.

All the structures were solved by direct methods (SHELXT-2014, 2015 [30] (for **5a**, **5b**) or XS [31] (for **5c**)) and refined by full-matrix least squares calculations on *F*^2^ (SHELXL-2015) [32] using the Olex2 program package [33].

**5a**: C_30_H_18_O_2_, orthorhombic, space group *pbca* (No. 61), *a* = 17.382(4) Å, *b* = 7.290(2) Å, *c* = 28.978(6) Å, *V* = 3672(1) Å^3^, ρ_calcd_ = 1.485 g/cm^3^, *Z* = 8, 925 unique reflections out of 4205 with *I* > 2σ (*I*), 291 parameters, 3.65º < θ < 15.71º, *R*_1_ = 0.1319, w*R*_2_ = 0.2925, GOF = 0.903.

**5b**: C_14.5_H_7_O, monoclinic, space group *P*2_1_/*m* (No. 11), *a* = 3.7712(7) Å, *b* = 15.097(3) Å, *c* = 14.845(3) Å, β = 113.312(3)°, *V* = 845.1(3) Å^3^, ρ_calcd_ = 1.550 g/cm^3^, *Z* = 4, 2296 unique reflections out of 2458 with *I* > 2σ(*I*), 166 parameters, 1.92º < θ < 30.03º, *R*_1_ = 0.0718, w*R*_2_= 0.2346, GOF = 1.153.

**5c**: C_29_H_14_O_2_, monoclinic, space group *P*2_1_/*c* (No. 14), *a* = 17.274(4) Å, *b* = 7.441(2) Å, *c* = 27.913(6) Å, β = 90.85(3)°, *V* = 3526(1) Å^3^, ρ_calcd_ = 1.486 g/cm^3^, *Z* = 4, 2351 unique reflections out of 7995 with *I* > 2σ(*I*), 253 parameters, 3.03º < θ < 27.37º, *R*_1_ = 0.1217, w*R*_2_ = 0.1498, GOF = 1.000.

CCDC 1981719 (**5a**), 1981720 (**5b**) and 1981721 (**5c**) contain the crystallographic data for this paper. These data can be obtained free of charge from The Cambridge Crystallographic Data Centre (https://www.ccdc.cam.ac.uk/).

## Supporting Information

File 1 CIF files of compounds **5a**–**c**.

File 2 ^1^H and ^13^C NMR data of **3a**, **3b**, **4a**, **4b** and **5a**–**c**, simulated UV–vis spectra of **5a**, **5b** and **5c**.
