# Supplementary material for: Synthesis of C70-fragment buckybowls bearing alkoxy substituents
Source: Beilstein J Org Chem. 2020 Apr 15;16:681–90. doi: 10.3762/bjoc.16.66 (PMC7176931; doi:10.3762/bjoc.16.66)

checkCIF/PLATON report

Structure factors have been supplied for datablock(s) Dimethoxy5a

THIS REPORT IS FOR GUIDANCE ONLY. IF USED AS PART OF A REVIEW PROCEDURE FOR PUBLICATION, IT SHOULD NOT REPLACE THE EXPERTISE OF AN EXPERIENCED CRYSTALLOGRAPHIC REFEREE.

No syntax errors found. CIF dictionary Interpreting this report

Datablock: Dimethoxy5a

```
Bond precision:  C-C = 0.0127 A          Wavelength=0.71073

Cell:             a=17.382(4)      b=7.2895(15)      c=28.978(6)
                  alpha=90         beta=90           gamma=90
Temperature:      150 K

                Calculated                 Reported
Volume          3671.7(14)                 3671.8(13)
Space group     P b c a                    P b c a
Hall group      -P 2ac 2ab                 -P 2ac 2ab
Moiety formula  C30 H18 O2                 C30 H18 O2
Sum formula     C30 H18 O2                 C30 H18 O2
Mr              410.44                     410.44
Dx,g cm-3       1.485                      1.485
Z               8                          8
Mu (mm-1)       0.092                      0.092
F000            1712.0                     1712.0
F000'           1712.75
h,k,lmax        22,9,37                    22,9,37
Nref            4217                       4205
Tmin,Tmax       0.989,0.999                0.656,1.000
Tmin'           0.980

Correction method= # Reported T Limits: Tmin=0.656 Tmax=1.000
AbsCorr = MULTI-SCAN

Data completeness= 0.997          Theta(max)= 27.483

R(reflections)= 0.1319( 925)      wR2(reflections)= 0.4008( 4205)

S = 0.903                   Npar= 291
```

The following ALERTS were generated. Each ALERT has the format
test-name_ALERT_alert-type_alert-level.
Click on the hyperlinks for more details of the test.

Alert level A

```
RINTA01_ALERT_3_A  The value of Rint is greater than 0.25
            Rint given   0.321
PLAT020_ALERT_3_A The Value of Rint is Greater Than 0.12 .........      0.321 Report
PLAT026_ALERT_3_A Ratio Observed / Unique Reflections (too) Low ..        22% Check
```

Alert level B

```
PLAT084_ALERT_3_B High wR2 Value (i.e. > 0.25) ...................       0.40 Report
PLAT340_ALERT_3_B Low Bond Precision on  C-C Bonds ...............    0.01269 Ang.
PLAT930_ALERT_2_B FCF-based   Twin Law ( 1 0 1)[ 3 0 1] Est.d BASF       0.58 Check
```

Alert level C

```
ABSTY02_ALERT_1_C  An _exptl_absorpt_correction_type has been given without
            a literature citation. This should be contained in the
            _exptl_absorpt_process_details field.
            Absorption correction given as multi-scan
PLAT082_ALERT_2_C High R1 Value ..................................       0.13 Report
PLAT213_ALERT_2_C Atom C12             has ADP max/min Ratio .....        3.3 oblate
PLAT234_ALERT_4_C Large Hirshfeld Difference O1       --C29      .       0.18 Ang.
PLAT234_ALERT_4_C Large Hirshfeld Difference C9       --C10      .       0.18 Ang.
PLAT234_ALERT_4_C Large Hirshfeld Difference C14      --C15      .       0.17 Ang.
PLAT241_ALERT_2_C High   MainMol Ueq as Compared to Neighbors of         O1 Check
PLAT241_ALERT_2_C High   MainMol Ueq as Compared to Neighbors of        C14 Check
PLAT242_ALERT_2_C Low    MainMol Ueq as Compared to Neighbors of        C12 Check
PLAT242_ALERT_2_C Low    MainMol Ueq as Compared to Neighbors of        C25 Check
PLAT334_ALERT_2_C Small Aver. Benzene C-C Dist C3       -C15             1.37 Ang.
PLAT334_ALERT_2_C Small Aver. Benzene C-C Dist C23      -C28             1.37 Ang.
PLAT363_ALERT_2_C Long   C(sp3)-C(sp2) Bond  C15      - C16      .       1.64 Ang.
PLAT906_ALERT_3_C Large K Value in the Analysis of Variance ......    453.055 Check
PLAT906_ALERT_3_C Large K Value in the Analysis of Variance ......      7.585 Check
PLAT906_ALERT_3_C Large K Value in the Analysis of Variance ......     57.595 Check
PLAT906_ALERT_3_C Large K Value in the Analysis of Variance ......      6.382 Check
PLAT906_ALERT_3_C Large K Value in the Analysis of Variance ......     23.678 Check
PLAT906_ALERT_3_C Large K Value in the Analysis of Variance ......      4.869 Check
PLAT906_ALERT_3_C Large K Value in the Analysis of Variance ......      9.395 Check
PLAT906_ALERT_3_C Large K Value in the Analysis of Variance ......      3.485 Check
PLAT906_ALERT_3_C Large K Value in the Analysis of Variance ......      5.364 Check
PLAT906_ALERT_3_C Large K Value in the Analysis of Variance ......      2.604 Check
PLAT906_ALERT_3_C Large K Value in the Analysis of Variance ......      3.119 Check
PLAT910_ALERT_3_C Missing # of FCF Reflection(s) Below Theta(Min).          5 Note
PLAT911_ALERT_3_C Missing FCF Refl Between Thmin & STh/L=    0.600          3 Report
```

Alert level G

```
PLAT003_ALERT_2_G Number of Uiso or Uij Restrained non-H Atoms ...         30 Report
PLAT012_ALERT_1_G No       _shelx_res_checksum Found in CIF ......     Please Check
PLAT072_ALERT_2_G SHELXL First  Parameter in WGHT  Unusually Large       0.15 Report
PLAT177_ALERT_4_G The CIF-Embedded .res File Contains DELU Records          5 Report
PLAT178_ALERT_4_G The CIF-Embedded .res File Contains SIMU Records          5 Report
PLAT187_ALERT_4_G The CIF-Embedded .res File Contains RIGU Records          1 Report
PLAT860_ALERT_3_G Number of Least-Squares Restraints .............        331 Note
PLAT870_ALERT_4_G ALERTS Related to Twinning Effects Suppressed ..          ! Info
PLAT883_ALERT_1_G No Info/Value for _atom_sites_solution_primary .     Please Do !
PLAT912_ALERT_4_G Missing # of FCF Reflections Above STh/L=  0.600          4 Note
PLAT931_ALERT_5_G CIFcalcFCF  Twin Law         [ 3 0 1] Est.d BASF       0.58 Check
```

3 **ALERT level A** = Most likely a serious problem - resolve or explain
3 **ALERT level B** = A potentially serious problem, consider carefully

```
26 ALERT level C = Check. Ensure it is not caused by an omission or oversight
11 ALERT level G = General information/check it is not something unexpected

 3 ALERT type 1 CIF construction/syntax error, inconsistent or missing data
12 ALERT type 2 Indicator that the structure model may be wrong or deficient
19 ALERT type 3 Indicator that the structure quality may be low
 8 ALERT type 4 Improvement, methodology, query or suggestion
 1 ALERT type 5 Informative message, check
```

It is advisable to attempt to resolve as many as possible of the alerts in all categories. Often the minor alerts point to easily fixed oversights, errors and omissions in your CIF or refinement strategy, so attention to these fine details can be worthwhile. In order to resolve some of the more serious problems it may be necessary to carry out additional measurements or structure refinements. However, the purpose of your study may justify the reported deviations and the more serious of these should normally be commented upon in the discussion or experimental section of a paper or in the "special_details" fields of the CIF. checkCIF was carefully designed to identify outliers and unusual parameters, but every test has its limitations and alerts that are not important in a particular case may appear. Conversely, the absence of alerts does not guarantee there are no aspects of the results needing attention. It is up to the individual to critically assess their own results and, if necessary, seek expert advice.

Publication of your CIF in IUCr journals

A basic structural check has been run on your CIF. These basic checks will be run on all CIFs submitted for publication in IUCr journals (*Acta Crystallographica*, *Journal of Applied Crystallography*, *Journal of Synchrotron Radiation*); however, if you intend to submit to *Acta Crystallographica Section C* or *E* or *IUCrData*, you should make sure that full publication checks are run on the final version of your CIF prior to submission.

Publication of your CIF in other journals

Please refer to the *Notes for Authors* of the relevant journal for any special instructions relating to CIF submission.

PLATON version of 22/12/2019; check.def file version of 13/12/2019

Datablock Dimethoxy5a - ellipsoid plot

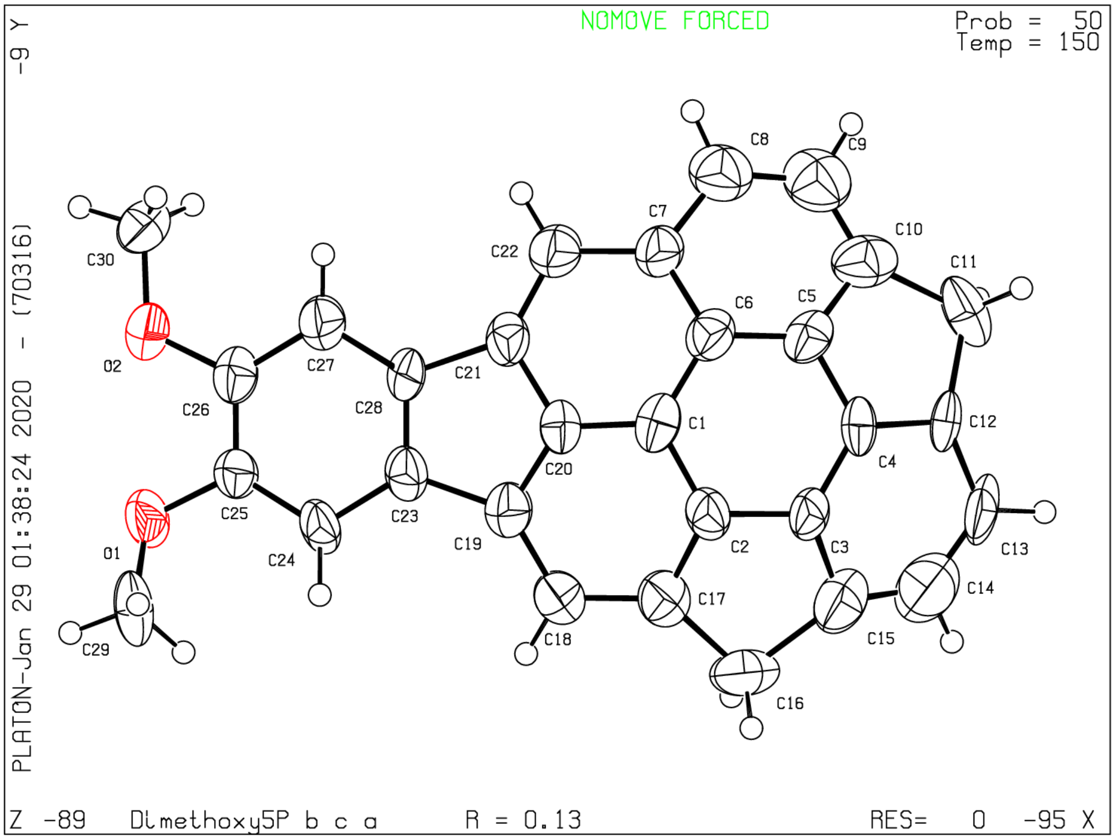

Supplement: File 1 — CIF files of compounds 5a–c. [file Beilstein_J_Org_Chem-16-681-s001.zip › Dimethoxy-5a.pdf]
