# Supplementary material for: Synthesis of C70-fragment buckybowls bearing alkoxy substituents
Source: Beilstein J Org Chem. 2020 Apr 15;16:681–90. doi: 10.3762/bjoc.16.66 (PMC7176931; doi:10.3762/bjoc.16.66)

checkCIF/PLATON report

Structure factors have been supplied for datablock(s) Dioxole5b

THIS REPORT IS FOR GUIDANCE ONLY. IF USED AS PART OF A REVIEW PROCEDURE FOR PUBLICATION, IT SHOULD NOT REPLACE THE EXPERTISE OF AN EXPERIENCED CRYSTALLOGRAPHIC REFEREE.

No syntax errors found. CIF dictionary Interpreting this report

Datablock: Dioxole5b

```
Bond precision:  C-C = 0.0032 A          Wavelength=0.70000

Cell:             a=3.7712(7)     b=15.097(3)       c=14.845(3)
                  alpha=90        beta=90.85(3)     gamma=90
Temperature:      150 K

                Calculated                 Reported
Volume          845.1(3)                   845.1(3)
Space group     P 21/m                     P 1 21/m 1
Hall group      -P 2yb                     -P 2yb
Moiety formula  C29 H14 O2                 C14.5 H7 O
Sum formula     C29 H14 O2                 C14.50 H7 O
Mr              394.40                     197.20
Dx,g cm-3       1.550                      1.550
Z               2                          4
Mu (mm-1)       0.092                      0.096
F000            408.0                      408.0
F000'           408.17
h,k,lmax        5,21,21                    5,21,20
Nref            2683                       2458
Tmin,Tmax
Tmin'

Correction method= Not given

Data completeness= 0.916          Theta(max)= 30.033

R(reflections)= 0.0718( 2296)     wR2(reflections)= 0.2360( 2458)

S = 1.153                   Npar= 166
```

The following ALERTS were generated. Each ALERT has the format
test-name_ALERT_alert-type_alert-level.
Click on the hyperlinks for more details of the test.

Alert level B

```
PLAT934_ALERT_3_B Number of (Iobs-Icalc)/Sigma(W) > 10 Outliers ..          2 Check
```

Alert level C

```
PLAT906_ALERT_3_C Large K Value in the Analysis of Variance ......      4.856 Check
PLAT911_ALERT_3_C Missing FCF Refl Between Thmin & STh/L=    0.600         17 Report
PLAT920_ALERT_1_C Theta(Max)  in CIF and FCF Differ by ...........       0.50 Degree
```

Alert level G

```
ABSMU01_ALERT_1_G  Calculation of _exptl_absorpt_correction_mu
                not performed for this radiation type.
PLAT012_ALERT_1_G No       _shelx_res_checksum Found in CIF ......     Please Check
PLAT042_ALERT_1_G Calc. and Reported MoietyFormula Strings  Differ     Please Check
PLAT045_ALERT_1_G Calculated and Reported Z Differ by a Factor ...       0.50 Check
PLAT072_ALERT_2_G SHELXL First  Parameter in WGHT  Unusually Large       0.10 Report
PLAT092_ALERT_4_G Check: Wavelength Given is not Cu,Ga,Mo,Ag,In Ka    0.70000 Ang.
PLAT230_ALERT_2_G Hirshfeld Test Diff for    C6        --C7B      .       10.5 s.u.
PLAT230_ALERT_2_G Hirshfeld Test Diff for    C8A       --C9       .        7.3 s.u.
PLAT230_ALERT_2_G Hirshfeld Test Diff for    C8B       --C9       .        8.5 s.u.
PLAT300_ALERT_4_G Atom Site Occupancy of C7A        Constrained at        0.5 Check
PLAT300_ALERT_4_G Atom Site Occupancy of C7B        Constrained at        0.5 Check
PLAT300_ALERT_4_G Atom Site Occupancy of C8A        Constrained at        0.5 Check
PLAT300_ALERT_4_G Atom Site Occupancy of C8B        Constrained at        0.5 Check
PLAT300_ALERT_4_G Atom Site Occupancy of H9BA       Constrained at        0.5 Check
PLAT300_ALERT_4_G Atom Site Occupancy of H9BB       Constrained at        0.5 Check
PLAT300_ALERT_4_G Atom Site Occupancy of H6AA       Constrained at        0.5 Check
PLAT300_ALERT_4_G Atom Site Occupancy of H6AB       Constrained at        0.5 Check
PLAT300_ALERT_4_G Atom Site Occupancy of H6B        Constrained at        0.5 Check
PLAT300_ALERT_4_G Atom Site Occupancy of H7A        Constrained at        0.5 Check
PLAT300_ALERT_4_G Atom Site Occupancy of H8B        Constrained at        0.5 Check
PLAT300_ALERT_4_G Atom Site Occupancy of H9A        Constrained at        0.5 Check
PLAT301_ALERT_3_G Main Residue  Disorder ..............(Resd  1  )        13% Note
PLAT398_ALERT_2_G Deviating  C-O-C    Angle From 120 for O1             104.3 Degree
PLAT720_ALERT_4_G Number of Unusual/Non-Standard Labels ..........          4 Note
PLAT910_ALERT_3_G Missing # of FCF Reflection(s) Below Theta(Min).          1 Note
PLAT912_ALERT_4_G Missing # of FCF Reflections Above STh/L=  0.600         88 Note
PLAT913_ALERT_3_G Missing # of Very Strong Reflections in FCF ....          1 Note
PLAT978_ALERT_2_G Number C-C Bonds with Positive Residual Density.          4 Info
PLAT992_ALERT_5_G Repd & Actual _reflns_number_gt Values Differ by          2 Check
```

---

0 **ALERT level A** = Most likely a serious problem - resolve or explain
1 **ALERT level B** = A potentially serious problem, consider carefully
3 **ALERT level C** = Check. Ensure it is not caused by an omission or oversight
29 **ALERT level G** = General information/check it is not something unexpected

5 ALERT type 1 CIF construction/syntax error, inconsistent or missing data
6 ALERT type 2 Indicator that the structure model may be wrong or deficient
6 ALERT type 3 Indicator that the structure quality may be low
15 ALERT type 4 Improvement, methodology, query or suggestion
1 ALERT type 5 Informative message, check

It is advisable to attempt to resolve as many as possible of the alerts in all categories. Often the minor alerts point to easily fixed oversights, errors and omissions in your CIF or refinement strategy, so attention to these fine details can be worthwhile. In order to resolve some of the more serious problems it may be necessary to carry out additional measurements or structure refinements. However, the purpose of your study may justify the reported deviations and the more serious of these should normally be commented upon in the discussion or experimental section of a paper or in the "special_details" fields of the CIF. checkCIF was carefully designed to identify outliers and unusual parameters, but every test has its limitations and alerts that are not important in a particular case may appear. Conversely, the absence of alerts does not guarantee there are no aspects of the results needing attention. It is up to the individual to critically assess their own results and, if necessary, seek expert advice.

Publication of your CIF in IUCr journals

A basic structural check has been run on your CIF. These basic checks will be run on all CIFs submitted for publication in IUCr journals (*Acta Crystallographica*, *Journal of Applied Crystallography*, *Journal of Synchrotron Radiation*); however, if you intend to submit to *Acta Crystallographica Section C* or *E* or *IUCrData*, you should make sure that full publication checks are run on the final version of your CIF prior to submission.

Publication of your CIF in other journals

Please refer to the *Notes for Authors* of the relevant journal for any special instructions relating to CIF submission.

PLATON version of 22/12/2019; check.def file version of 13/12/2019

Datablock Dioxole5b - ellipsoid plot

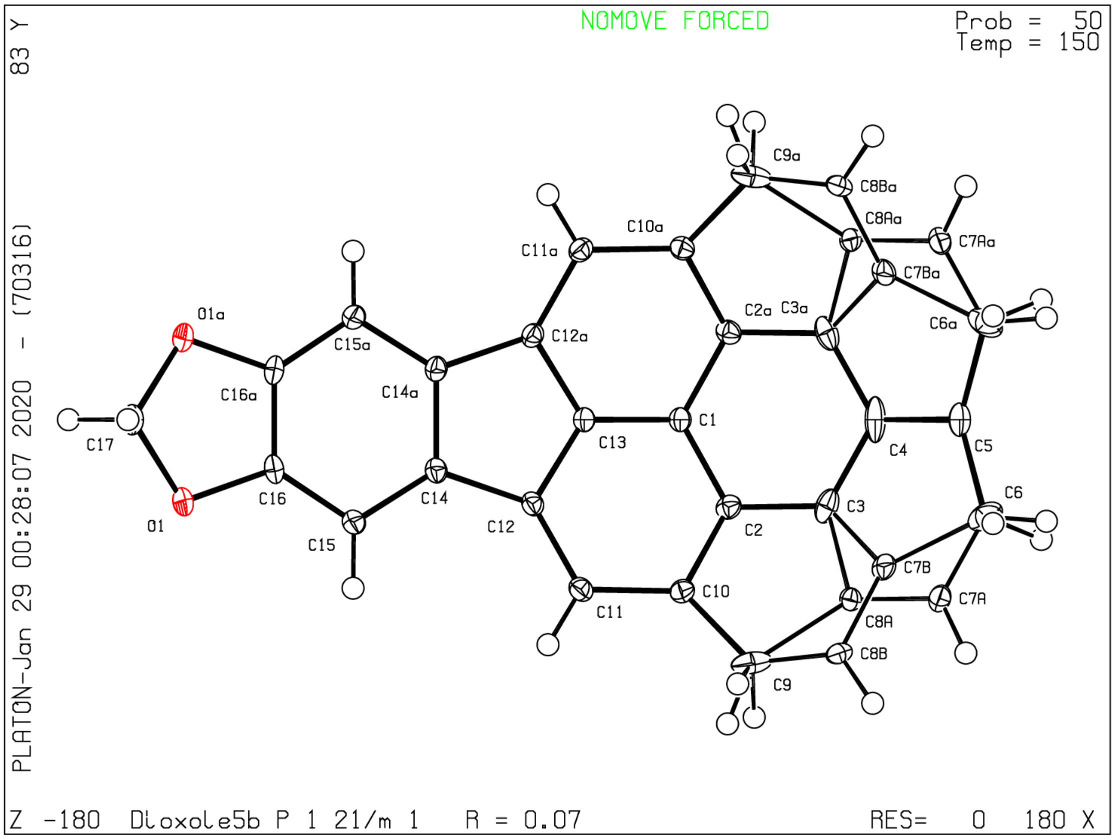

Supplement: File 1 — CIF files of compounds 5a–c. [file Beilstein_J_Org_Chem-16-681-s001.zip › Dioxole-5b.pdf]
