# Supplementary material for: Synthesis of C70-fragment buckybowls bearing alkoxy substituents
Source: Beilstein J Org Chem. 2020 Apr 15;16:681–90. doi: 10.3762/bjoc.16.66 (PMC7176931; doi:10.3762/bjoc.16.66)

checkCIF/PLATON report

Structure factors have been supplied for datablock(s) Dioxole5c

THIS REPORT IS FOR GUIDANCE ONLY. IF USED AS PART OF A REVIEW PROCEDURE FOR PUBLICATION, IT SHOULD NOT REPLACE THE EXPERTISE OF AN EXPERIENCED CRYSTALLOGRAPHIC REFEREE.

No syntax errors found. CIF dictionary Interpreting this report

Datablock: Dioxole5c

```
Bond precision:  C-C = 0.0084 A          Wavelength=0.71073

Cell:            a=17.274(4)     b=7.4408(15)       c=27.913(6)
                 alpha=90        beta=100.62(3)     gamma=90
Temperature:     150 K

                Calculated                Reported
Volume          3526.3(14)                3526.2(13)
Space group     P 21/c                    P 1 21/c 1
Hall group      -P 2ybc                   -P 2ybc
Moiety formula  C29 H14 O2                C29 H14 O2
Sum formula     C29 H14 O2                C29 H14 O2
Mr              394.40                    394.40
Dx,g cm-3       1.486                     1.486
Z               8                         8
Mu (mm-1)       0.092                     0.092
F000            1632.0                    1632.0
F000'           1632.73
h,k,lmax        22,9,36                   22,9,35
Nref            8104                      7995
Tmin,Tmax       0.997,0.997               0.544,1.000
Tmin'           0.984

Correction method= # Reported T Limits: Tmin=0.544 Tmax=1.000
AbsCorr = MULTI-SCAN

Data completeness= 0.987          Theta(max)= 27.485

R(reflections)= 0.1217( 2351)     wR2(reflections)= 0.2096( 7995)

S = 1.000                   Npar= 559
```

The following ALERTS were generated. Each ALERT has the format
test-name_ALERT_alert-type_alert-level.
Click on the hyperlinks for more details of the test.

Alert level A

```
PLAT026_ALERT_3_A Ratio Observed / Unique Reflections (too) Low ..        29% Check
```

Alert level B

```
RINTA01_ALERT_3_B  The value of Rint is greater than 0.18
            Rint given   0.188
PLAT020_ALERT_3_B The Value of Rint is Greater Than 0.12 .........      0.188 Report
PLAT230_ALERT_2_B Hirshfeld Test Diff for    C23      --C28      .        7.4 s.u.
```

Alert level C

```
ABSTY02_ALERT_1_C  An _exptl_absorpt_correction_type has been given without
            a literature citation. This should be contained in the
            _exptl_absorpt_process_details field.
            Absorption correction given as multi-scan
PLAT082_ALERT_2_C High R1 Value ..................................       0.12 Report
PLAT230_ALERT_2_C Hirshfeld Test Diff for    O2       --C27      .        5.6 s.u.
PLAT230_ALERT_2_C Hirshfeld Test Diff for    C3       --C14      .        5.8 s.u.
PLAT230_ALERT_2_C Hirshfeld Test Diff for    C10      --C11      .        5.6 s.u.
PLAT230_ALERT_2_C Hirshfeld Test Diff for    C55      --C56      .        5.3 s.u.
PLAT234_ALERT_4_C Large Hirshfeld Difference C39      --C40      .       0.17 Ang.
PLAT234_ALERT_4_C Large Hirshfeld Difference C43      --C44      .       0.16 Ang.
PLAT234_ALERT_4_C Large Hirshfeld Difference C45      --C46      .       0.16 Ang.
PLAT234_ALERT_4_C Large Hirshfeld Difference C46      --C47      .       0.18 Ang.
PLAT234_ALERT_4_C Large Hirshfeld Difference C52      --C57      .       0.16 Ang.
PLAT241_ALERT_2_C High   MainMol Ueq as Compared to Neighbors of         O4 Check
PLAT340_ALERT_3_C Low Bond Precision on  C-C Bonds ...............    0.00843 Ang.
PLAT906_ALERT_3_C Large K Value in the Analysis of Variance ......    131.081 Check
PLAT906_ALERT_3_C Large K Value in the Analysis of Variance ......      3.001 Check
PLAT906_ALERT_3_C Large K Value in the Analysis of Variance ......     21.787 Check
PLAT906_ALERT_3_C Large K Value in the Analysis of Variance ......      2.828 Check
PLAT906_ALERT_3_C Large K Value in the Analysis of Variance ......      7.194 Check
PLAT906_ALERT_3_C Large K Value in the Analysis of Variance ......      2.551 Check
PLAT906_ALERT_3_C Large K Value in the Analysis of Variance ......      3.764 Check
PLAT906_ALERT_3_C Large K Value in the Analysis of Variance ......      2.453 Check
PLAT910_ALERT_3_C Missing # of FCF Reflection(s) Below Theta(Min).         10 Note
PLAT911_ALERT_3_C Missing FCF Refl Between Thmin & STh/L=    0.600         32 Report
PLAT978_ALERT_2_C Number C-C Bonds with Positive Residual Density.          0 Info
```

Alert level G

```
PLAT003_ALERT_2_G Number of Uiso or Uij Restrained non-H Atoms ...          6 Report
PLAT012_ALERT_1_G No       _shelx_res_checksum Found in CIF ......     Please Check
PLAT177_ALERT_4_G The CIF-Embedded .res File Contains DELU Records          1 Report
PLAT178_ALERT_4_G The CIF-Embedded .res File Contains SIMU Records          1 Report
PLAT187_ALERT_4_G The CIF-Embedded .res File Contains RIGU Records          1 Report
PLAT398_ALERT_2_G Deviating  C-O-C    Angle From 120 for O1             103.5 Degree
PLAT398_ALERT_2_G Deviating  C-O-C    Angle From 120 for O2             105.2 Degree
PLAT398_ALERT_2_G Deviating  C-O-C    Angle From 120 for O3             105.8 Degree
PLAT398_ALERT_2_G Deviating  C-O-C    Angle From 120 for O4             106.4 Degree
PLAT860_ALERT_3_G Number of Least-Squares Restraints .............         51 Note
PLAT883_ALERT_1_G No Info/Value for _atom_sites_solution_primary .     Please Do !
PLAT912_ALERT_4_G Missing # of FCF Reflections Above STh/L=  0.600         59 Note
```

1 **ALERT level A** = Most likely a serious problem - resolve or explain
3 **ALERT level B** = A potentially serious problem, consider carefully
24 **ALERT level C** = Check. Ensure it is not caused by an omission or oversight
12 **ALERT level G** = General information/check it is not something unexpected

```
 3 ALERT type 1 CIF construction/syntax error, inconsistent or missing data
13 ALERT type 2 Indicator that the structure model may be wrong or deficient
15 ALERT type 3 Indicator that the structure quality may be low
 9 ALERT type 4 Improvement, methodology, query or suggestion
 0 ALERT type 5 Informative message, check
```

It is advisable to attempt to resolve as many as possible of the alerts in all categories. Often the minor alerts point to easily fixed oversights, errors and omissions in your CIF or refinement strategy, so attention to these fine details can be worthwhile. In order to resolve some of the more serious problems it may be necessary to carry out additional measurements or structure refinements. However, the purpose of your study may justify the reported deviations and the more serious of these should normally be commented upon in the discussion or experimental section of a paper or in the "special_details" fields of the CIF. checkCIF was carefully designed to identify outliers and unusual parameters, but every test has its limitations and alerts that are not important in a particular case may appear. Conversely, the absence of alerts does not guarantee there are no aspects of the results needing attention. It is up to the individual to critically assess their own results and, if necessary, seek expert advice.

Publication of your CIF in IUCr journals

A basic structural check has been run on your CIF. These basic checks will be run on all CIFs submitted for publication in IUCr journals (*Acta Crystallographica*, *Journal of Applied Crystallography*, *Journal of Synchrotron Radiation*); however, if you intend to submit to *Acta Crystallographica Section C* or *E* or *IUCrData*, you should make sure that full publication checks are run on the final version of your CIF prior to submission.

Publication of your CIF in other journals

Please refer to the *Notes for Authors* of the relevant journal for any special instructions relating to CIF submission.

PLATON version of 22/12/2019; check.def file version of 13/12/2019

Datablock Dioxole5c - ellipsoid plot

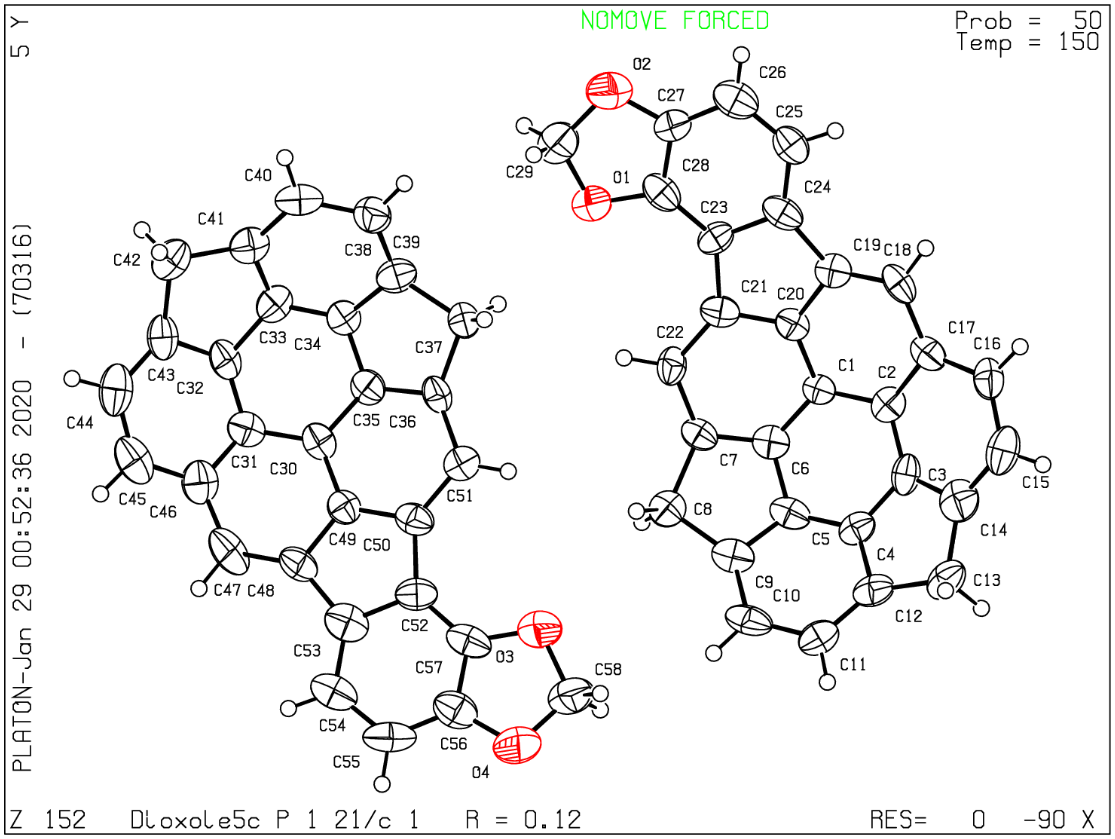

Supplement: File 1 — CIF files of compounds 5a–c. [file Beilstein_J_Org_Chem-16-681-s001.zip › Dioxole-5c.pdf]
