# Supplementary material for: Synthesis of C70-fragment buckybowls bearing alkoxy substituents
Source: Beilstein J Org Chem. 2020 Apr 15;16:681–90. doi: 10.3762/bjoc.16.66 (PMC7176931; doi:10.3762/bjoc.16.66)
Supplement: File 2 — 1H and 13C NMR data of 3a, 3b, 4a, 4b and 5a–c, simulated UV–vis spectra of 5a, 5b and 5c. [file Beilstein_J_Org_Chem-16-681-s002.pdf]

BEILSTEIN JOURNAL OF ORGANIC CHEMISTRY

# Supporting Information

for

# Synthesis of $C_{70}$-fragment buckybowls bearing alkoxy substituents

Yumi Yakiyama, Shota Hishikawa and Hidehiro Sakurai

*Beilstein J. Org. Chem.* **2020,** *16,* 681–690. doi:10.3762/bjoc.16.66

## $^{1}H$ and $^{13}C$ NMR data of 3a, 3b, 4a, 4b and 5a–c, simulated UV–vis spectra of 5a, 5b and 5c

License and Terms: This is a supporting information file under the terms of the Creative Commons Attribution License (http://creativecommons.org/licenses/by/4.0). Please note that the reuse, redistribution and reproduction in particular requires that the authors and source are credited.
The license is subject to the *Beilstein Journal of Organic Chemistry* terms and conditions: (https://www.beilstein-journals.org/bjoc)

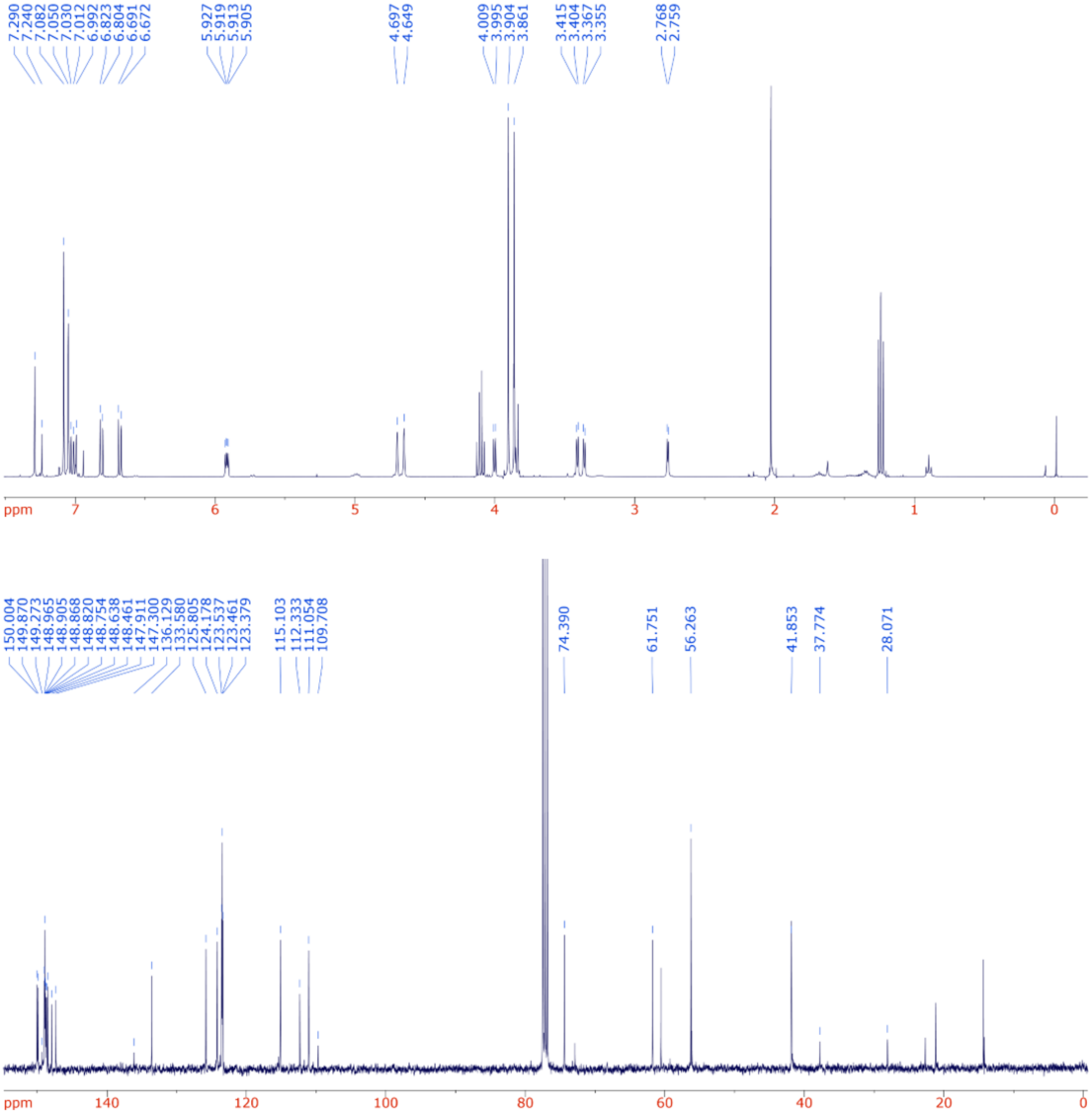


Figure S1. $^{1}H$ and $^{13}C$ NMR spectra of **3a**.

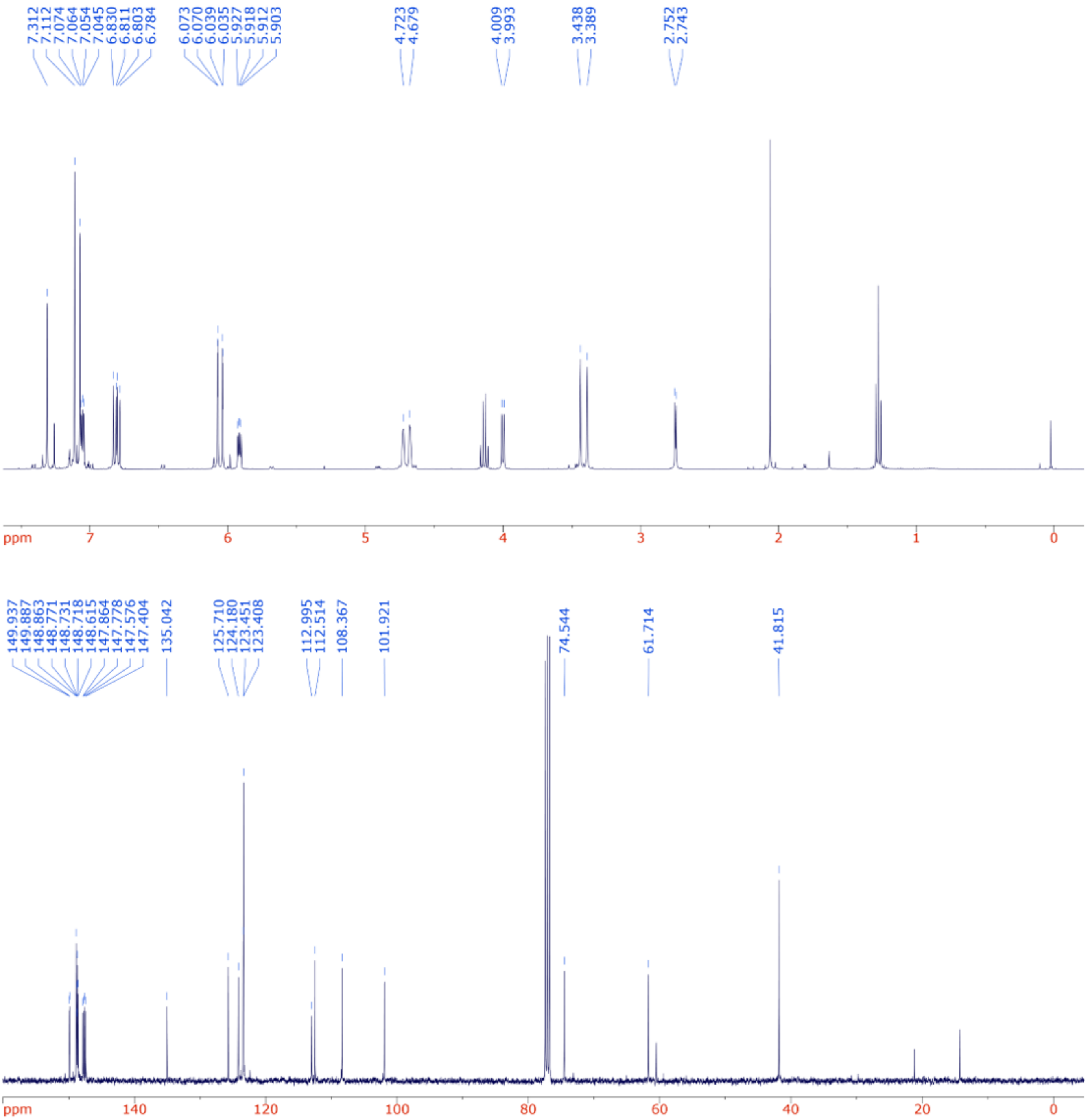


Figure S2. $^{1}H$ and $^{13}C$ NMR spectra of **3b**.

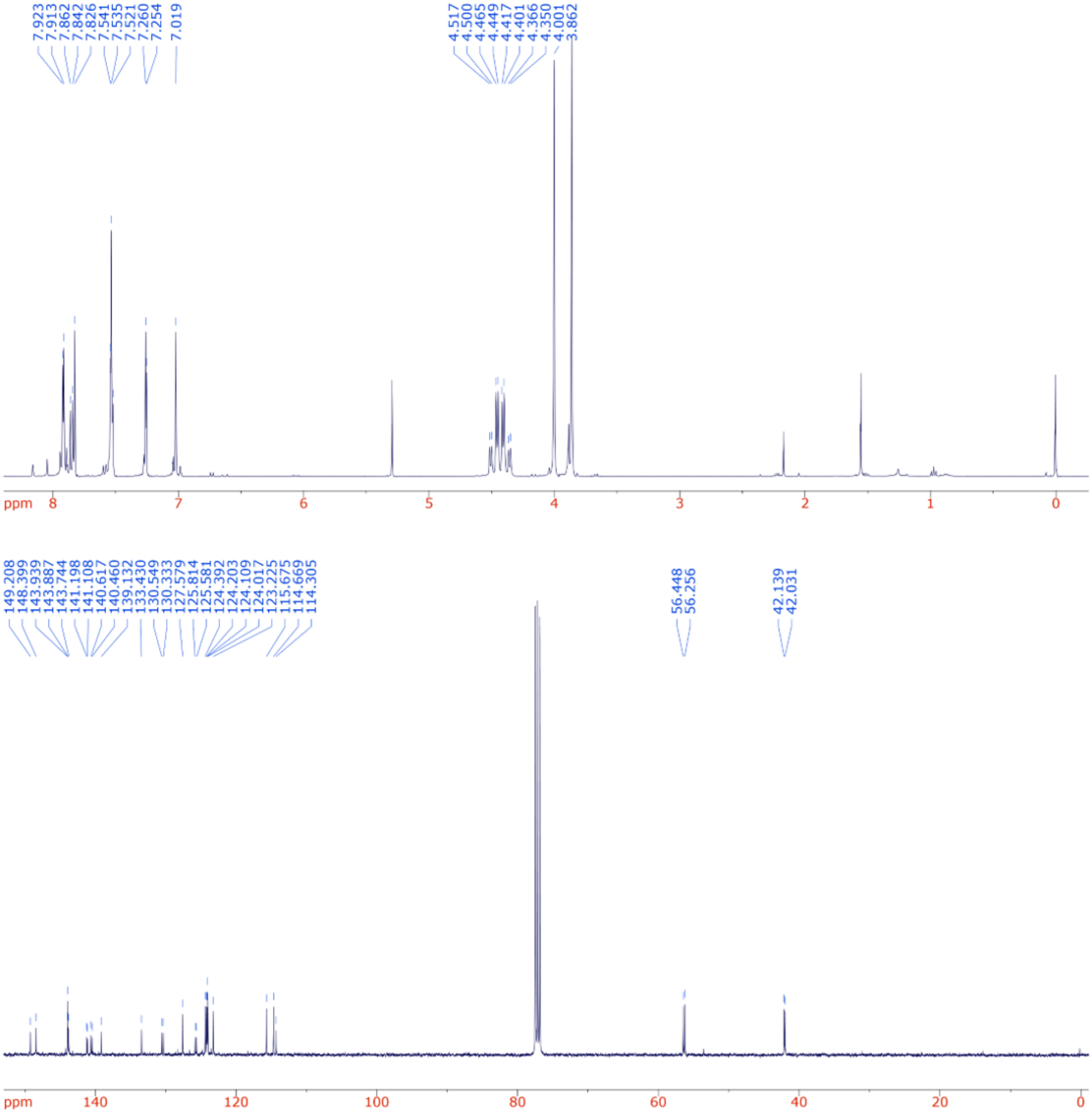


Figure S3. $^{1}H$ and $^{13}C$ NMR spectra of **4a**.

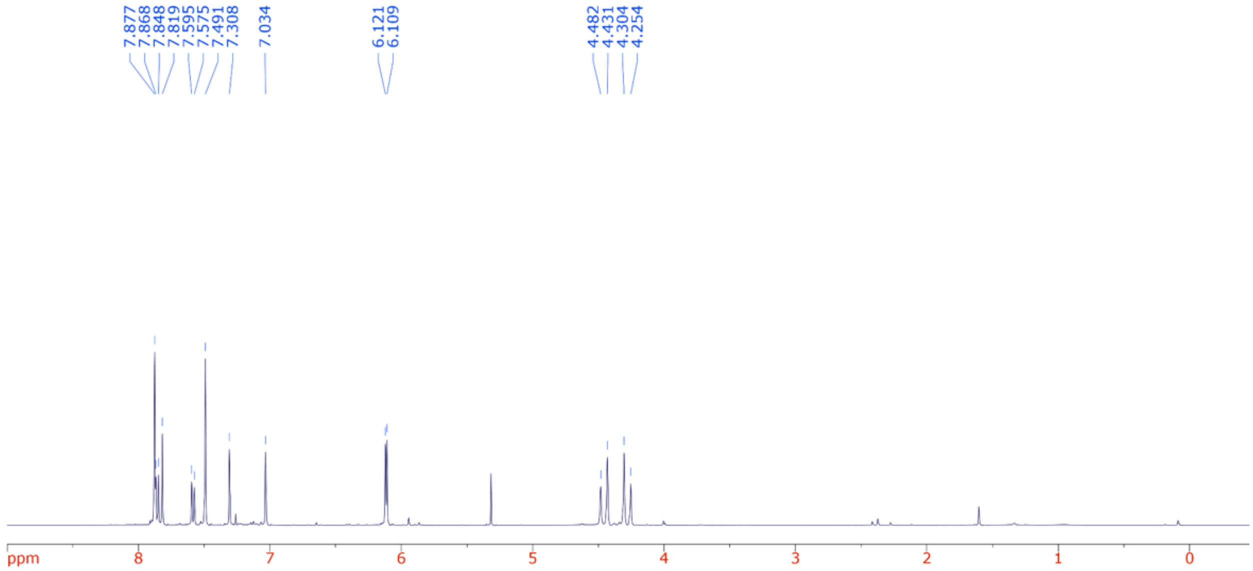


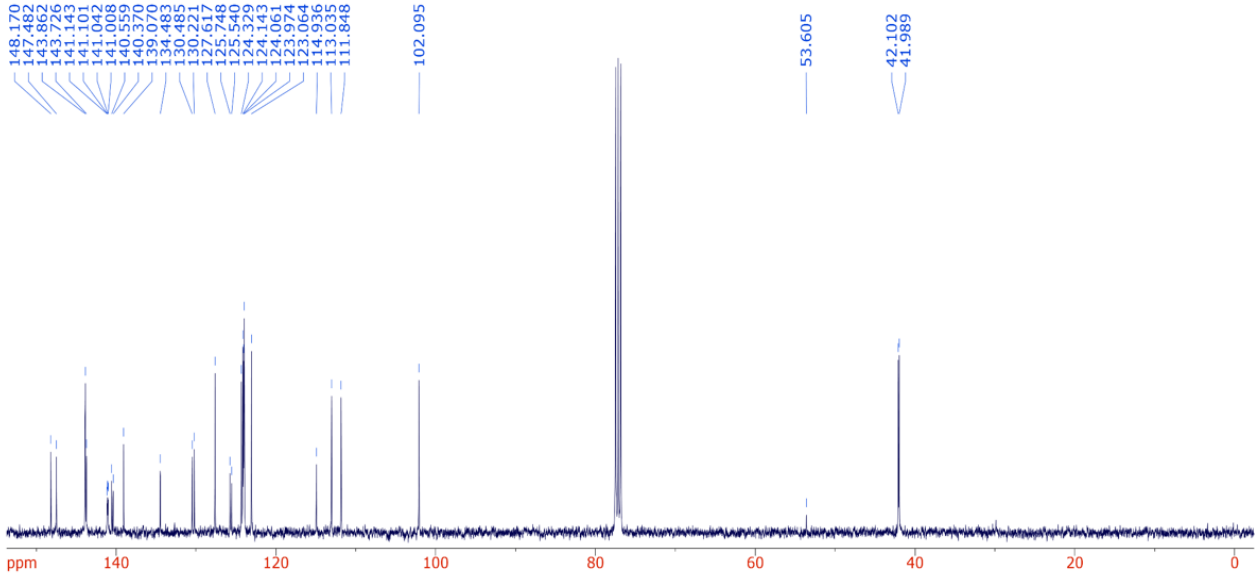


Figure S4. $^{1}$H and $^{13}$C NMR spectra of **4b**.

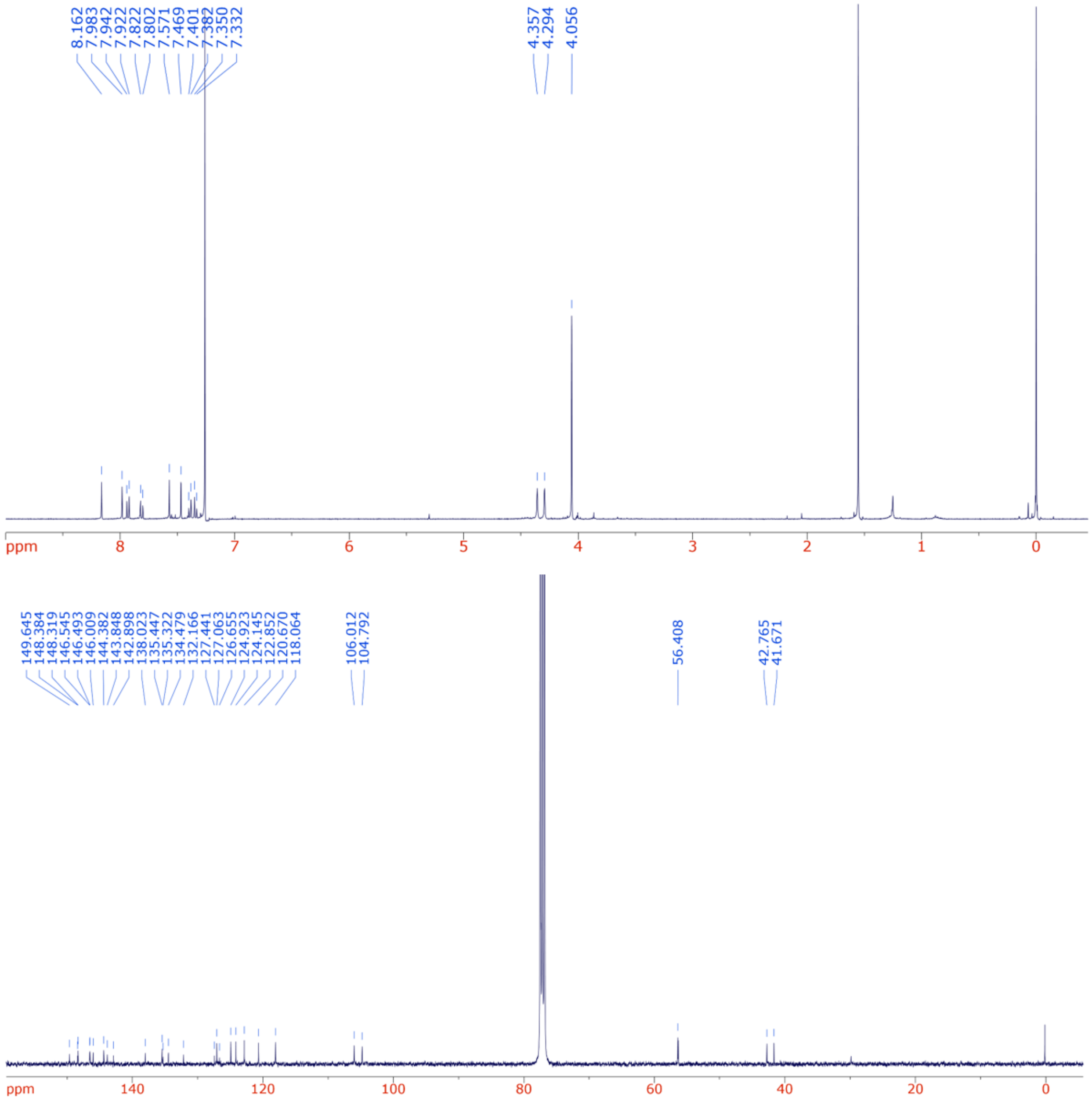


Figure S5. $^{1}H$ and $^{13}C$ NMR spectra of **5a**.

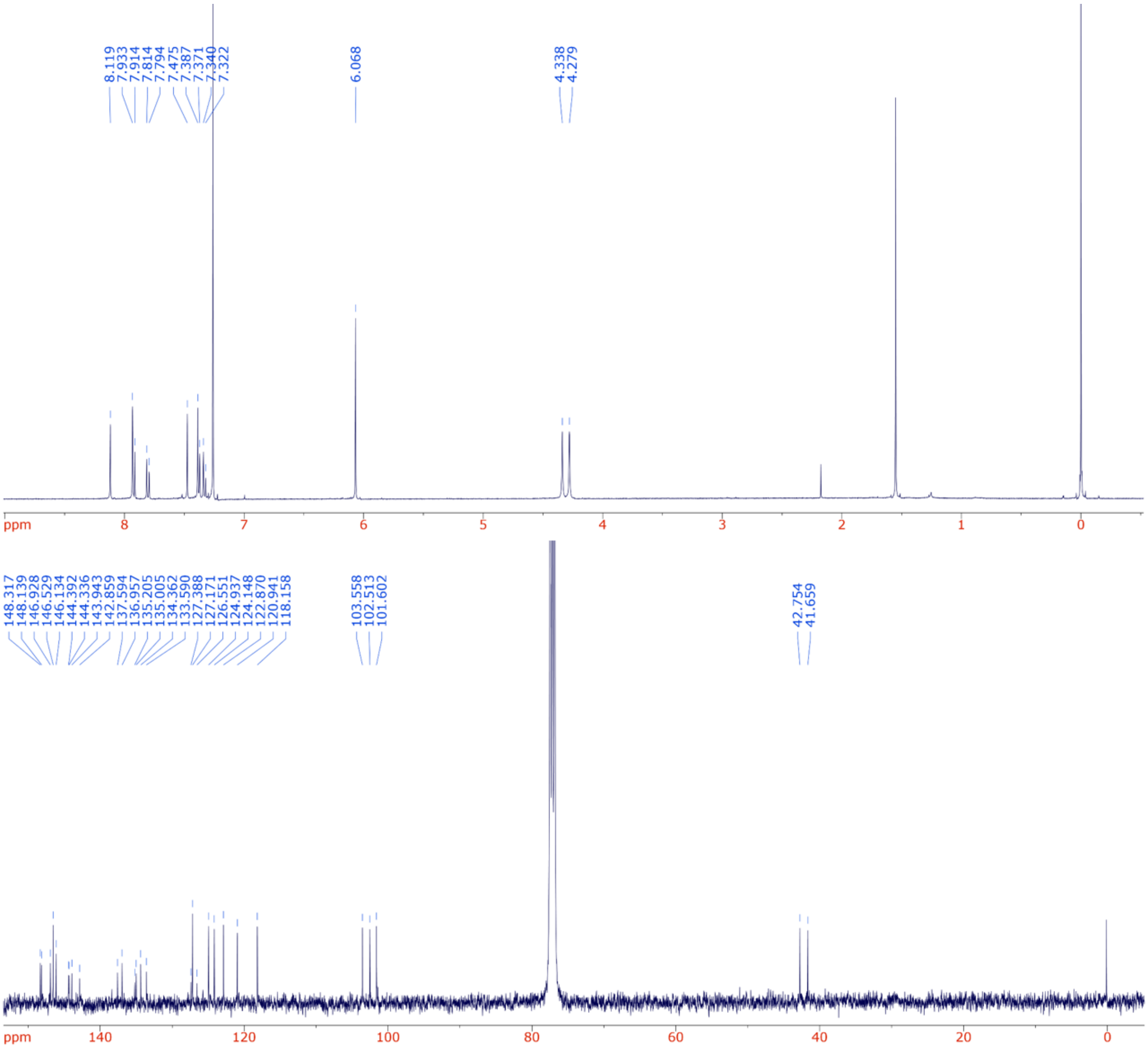


Figure S6. $^1$H and $^{13}$C NMR spectra of **5b**.

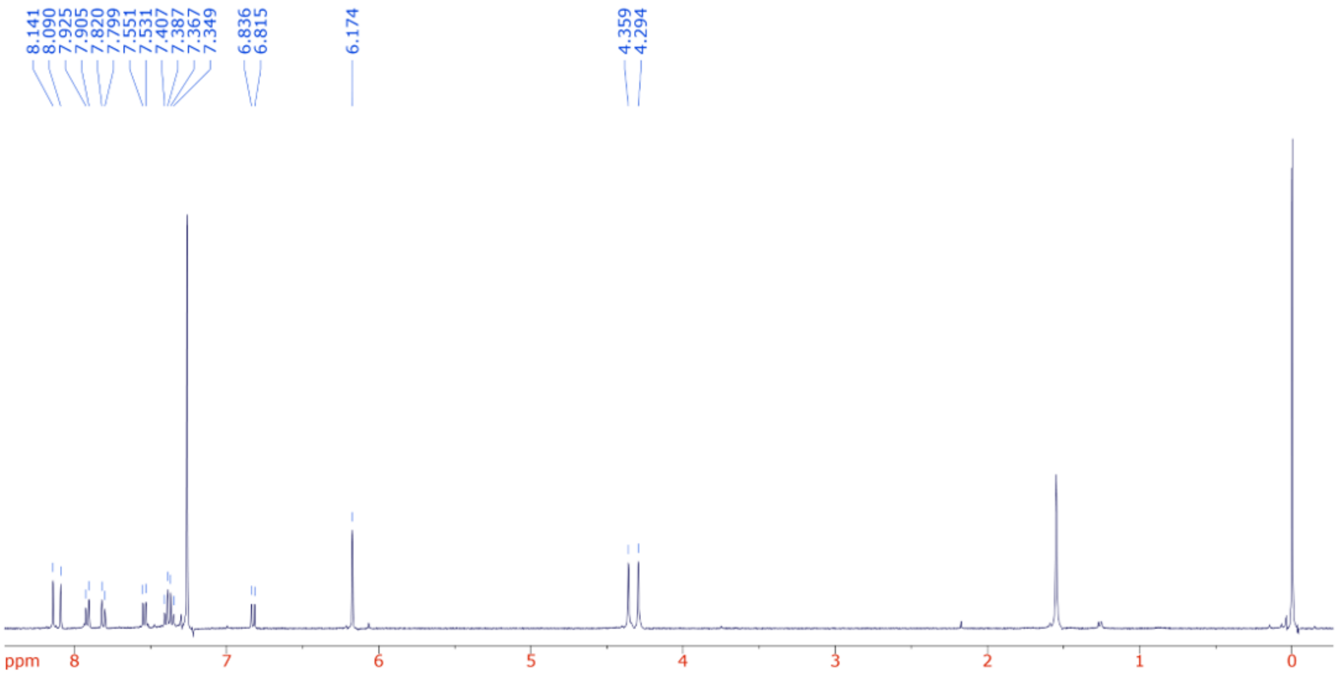


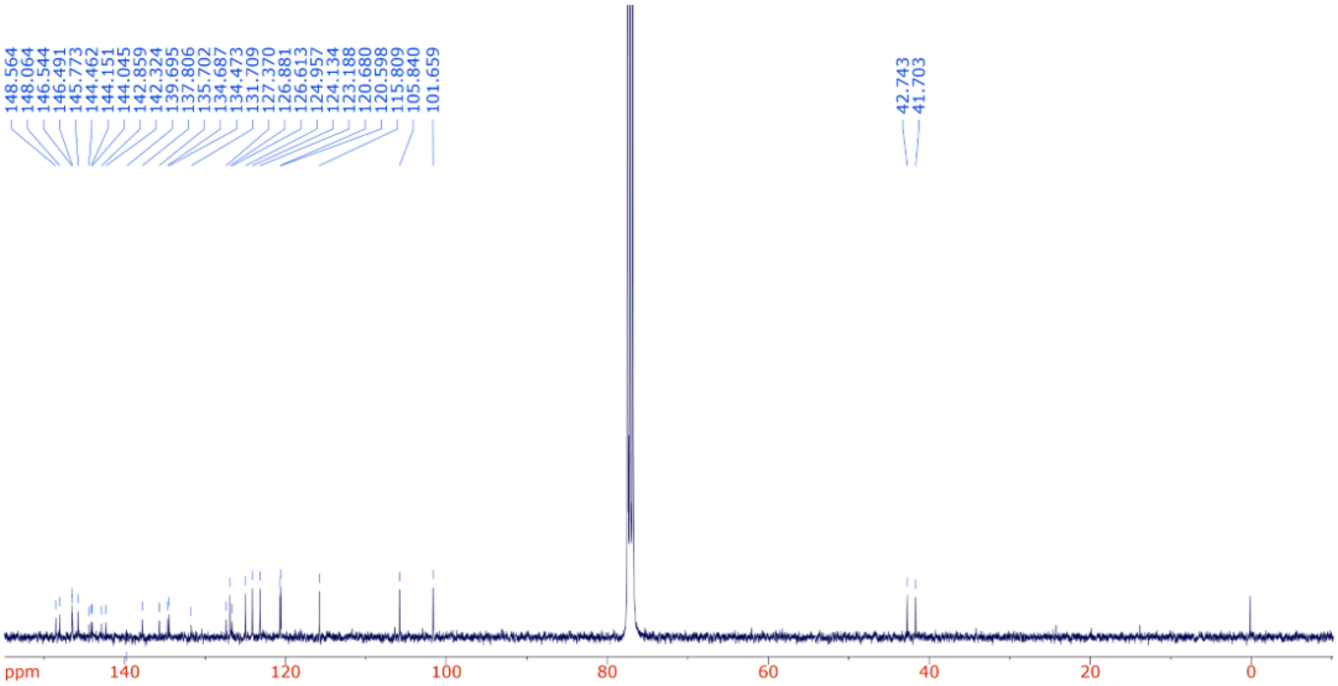


Figure S7. $^{1}H$ and $^{13}C$ NMR spectra of **5c**.

## 2. Computational data

All the theoretical calculations were conducted by Gaussian09.[S1] Calculation of UV-vis spectra of **5a**, **5b** and **5c** was performed by TD-DFT (time dependent density functional theory) at the CAM-B3LYP/6-31G+(d,p) level of theory with using the optimized structures from single crystal X-ray analysis data.

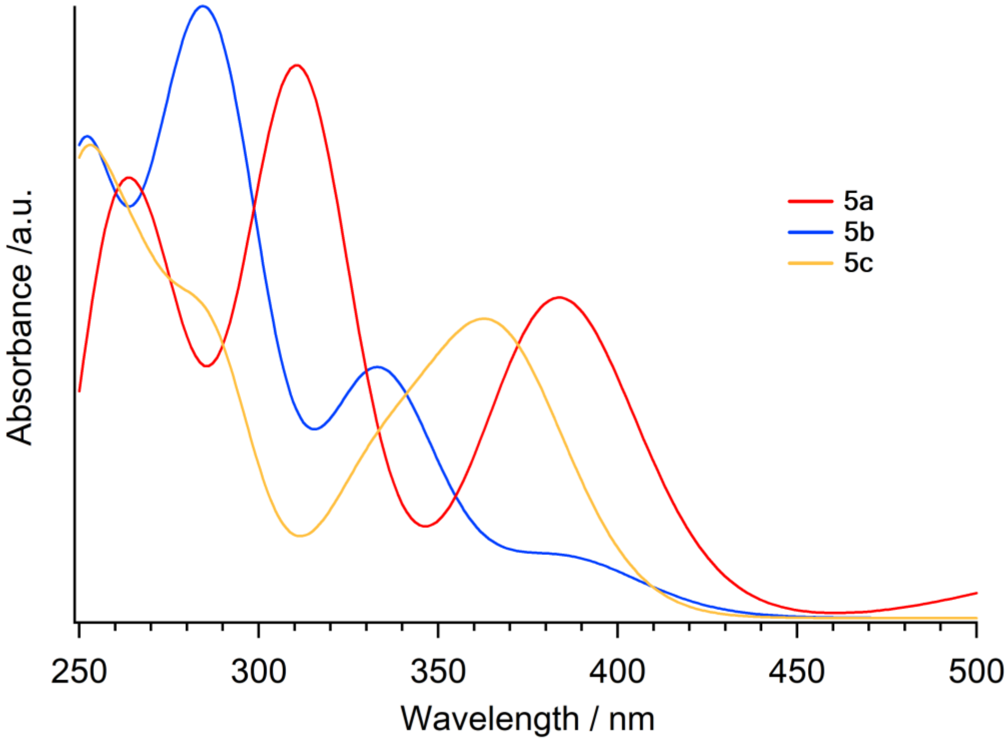


Figure S8. Simulated UV-vis spectra of **5a**, **5b** and **5c**.

## Reference

S1. M. J. Frisch, G. W. Trucks, H. B. Schlegel,G. E. Scuseria, M. A. Robb, J. R. Cheeseman, G. Scalmani, V. Barone, B. Mennucci, G. A. Petersson, H. Nakatsuji, M. Maricato, X. Li, H. P. Hratchian, A. F. Izmaylov, J. Bloino, G. Zheng, I. L. Sonnenberg, M. Hada, M. Ehara, K. Toyota, R. Fukuda, J. Hasegawa, M. Ishida, T. Nakajima,Y. Honda, O. Kitao, H. Nakai, T. Vreven, J. A. Montgomery, J. E. Peralta, F. Ogliaro, M. Bearpark, J. J. Heyd, E. Brothers, K. N. Kudin,V. N. Staroverov, R. Kobayashi, J. Normand, K. Raghavachari, A. Rendell, J. C. Burant, S. S. Iyengar, J. Tomasi, M. Cossi, N. Rega, N. J. Millam, M. Klene, J. E. Knox, J. B. Cross, V. Bakken, C. Adamo, J. Jaramillo, R. Gomperts, R. E. Stratmann, O. Yazyev, A. J. Austin, R. Cammi, C. Pomelli, J. W. Ochterski, R. L. Martin, K. Morokuma, V. G. Zakrzewski, G. A. Voth, P. Salvador, J. J. Dannenberg, S. Dapprich, A. D. Daniels, Ö. Farkas, J. B. Foresman, J. V. Ortiz, J. Cioslowski, D. J. Fox, Gaussian09, Revision B.01; Gaussian Inc.: Wallingford, CT, 2010.
